# Biophysical and structural studies reveal marginal stability of a crucial hydrocarbon biosynthetic enzyme acyl ACP reductase

**DOI:** 10.1038/s41598-021-91232-0

**Published:** 2021-06-08

**Authors:** Ashima Sharma, Tabinda Shakeel, Mayank Gupta, Girish H. Rajacharya, Syed Shams Yazdani

**Affiliations:** 1 Microbial Engineering Group, International Centre for Genetic Engineering and Biotechnology, New Delhi, India; 2 DBT-ICGEB Centre for Advanced Bioenergy Research, International Centre for Genetic Engineering and Biotechnology, New Delhi, India

**Keywords:** Biophysics, Biotechnology

## Abstract

Acyl-ACP reductase (AAR) is one of the two key cyanobacterial enzymes along with aldehyde deformylating oxygenase (ADO) involved in the synthesis of long-chain alkanes, a drop-in biofuel. The enzyme is prone to aggregation when expressed in *Escherichia coli*, leading to varying alkane levels. The present work attempts to investigate the crucial structural aspects of AAR protein associated with its stability and folding. Characterization by dynamic light scattering experiment and intact mass spectrometry revealed that recombinantly expressed AAR in *E. coli* existed in multiple-sized protein particles due to diverse lipidation. Interestingly, while thermal- and urea-based denaturation of AAR showed 2-state unfolding transition in circular dichroism and intrinsic fluorescent spectroscopy, the unfolding process of AAR was a 3-state pathway in GdnHCl solution suggesting that the protein milieu plays a significant role in dictating its folding. Apparent standard free energy $\left( {\Delta {\text{G}}_{{{\text{NU}}}}^{{{\text{H}}_{2} {\text{O}}}} } \right)$ of ~ 4.5 kcal/mol for the steady-state unfolding of AAR indicated borderline stability of the protein. Based on these evidences, we propose that the marginal stability of AAR are plausible contributing reasons for aggregation propensity and hence the low catalytic activity of the enzyme when expressed in *E. coli* for biofuel production. Our results show a path for building superior biocatalyst for higher biofuel production.

## Introduction

The emerging unavailability of fossil fuels, heightened levels of pollutants, and greenhouse gas emissions have directed attention towards the development of safe and renewable energy sources^1,2^. Biofuels have the potential to provide a sustainable cost-effective alternative for petroleum-based hydrocarbons. High energy density and compatibility of the long-chain alk(a/e)ne with the existing engines and infrastructure furthers its demand as an effective and ideal candidate for drop-in biofuels^2,3^.

Numerous reports are available showing ability of various microorganisms, primarily cyanobacteria, to produce long-chain alk(a/e)nes from fatty acid precursors^4–9^. Cyanobacterial alkane producing pathway consists of two slow-acting, water-soluble enzymes, namely Acyl-ACP reductase (AAR) (UniProtKB Q54765) and aldehyde deformylating oxygenase (ADO) (UniProtKB Q54764)^10^. AAR catalyzes NADPH-dependent reduction of fatty acyl ACP/CoA to aldehydes which then get converted into one carbon less (C_n-1_) alkanes by ADO^10–12^. To date, only ~ 34% (425 mg/L) of the maximum theoretical alkane level and 2.5 g/L alkane during fed-batch fermentation have been achieved in the *E. coli* host which is still incompetent for use at commercial scale^8^. One of the bottlenecks associated is the low catalytic efficiency of the enzymes involved^11–13^.

**Figure 1:**
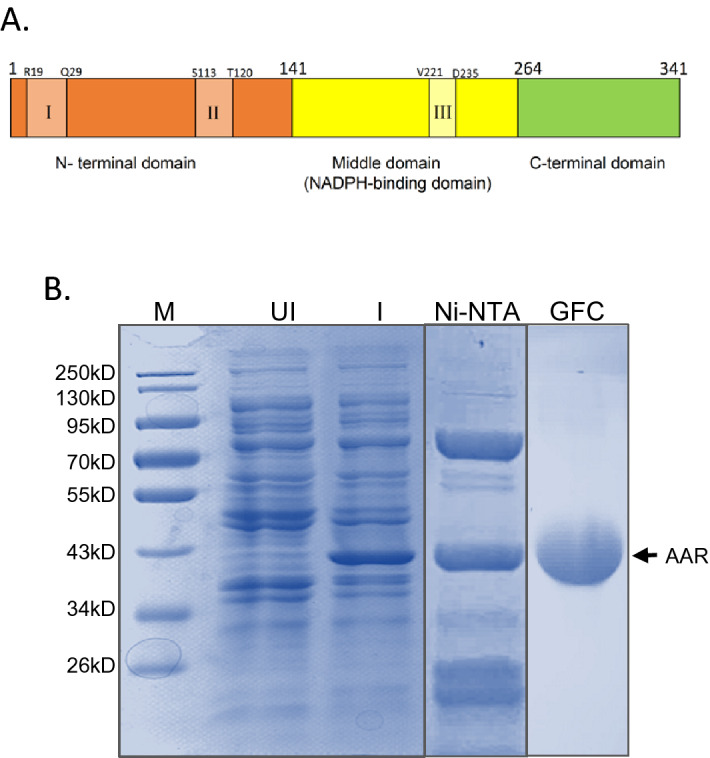
(**A**) Domain organization of AAR. I, II, III represents intrinsic flexible regions in the respective domains. (**B**) Expression and purification of AAR. 12% SDS PAGE showing expression of AAR in E. coli cells. Lane M, medium-range protein molecular weight marker; Lane UI, uninduced M15 (pQE30 AAR) E. coli cells; Lane I, Induced M15 (pQE30AAR) cells; Lane Ni–NTA, Ni–NTA chromatography purified AAR; Lane GFC, GFC purified and concentrated AAR. The represented lanes here are extracted from full gel images shown in Figure S1.

AAR is a key enzyme of the pathway and is prone to aggregation and the majority of protein forms inclusion bodies when expressed in *E. coli* ^11^*.* Interestingly, the alkane level varies among different cyanobacteria strains with variable soluble AAR levels^14^. AAR has been reported recently to contain three flexible regions possessing intrinsic flexibility in the N-terminal and mid-domain of the structure (Fig. 1A)^15^. AAR consists of three domains: the N-terminal domain (NTD, residues 1–130), the middle domain (mid-domain, residues 131–264) with a conserved dinucleotide recognition loop for NADPH binding, and the C-terminal domain (CTD, residues 265–341)^15^. Residue C294, which is strictly conserved in AAR homologs, is located at the center of the AAR molecule^15^.

Studies have been carried out exploring the functional characterization of AAR and gaining structural insights into the mechanistic aspects of AAR-ADO function^10,12,14,15^. Interestingly, Gao et al. have recently reported that expressed AAR in *E. coli* was unable to form stable crystals as such and only the apo-AAR or the complex with ADO and ligand yielded crystals for the study^15^. Therefore, gaining deep insights into the structural aspects of the expressed AAR protein is an irrefutable concern which needs to be addressed. Proper folding, conformation, and stability are very crucial factors to form functionally active AAR protein. Reports are available where ADO, the consecutive enzyme in the pathway, has exhibited remarkable enhanced activity upon improvement in the stability of the protein^2,16^.

Therefore, it is imperative to understand the stability and folding of the protein, given the immense biosynthetic potential of AAR in industries for hydrocarbon production. However, so far there are no reports available regarding the conformational and thermodynamics stability characterization of AAR, which are among the major goals for industrially relevant proteins that are likely to be exposed to extreme conditions such as high temperature, presence of co-solvents, low pH, etc. These environmental conditions impact the stability and integrity of the protein via perturbation of the intramolecular bonds. A natively folded protein can withstand the crowded biological environment and perform the assigned role. The failure to attain the biological conformation results in misfolded and inactive protein encompassing undesirable features. For proteins of biotechnological significance like AAR, understanding of environmental factors that dictate stability and folding will be important for better production of the functional recombinant enzyme at industrial scale.

In the present study, we purified the heterologously expressed AAR in *E. coli* and performed detailed in vitro physico-chemical characterization of AAR to gain insights into elucidating the thermodynamic parameters and conformational stability of the protein. We studied the equilibrium unfolding pathway of AAR by carrying out chemical denaturant-, and heat-mediated unfolding experiments, and the unfolding data was used for the calculation of thermodynamic stability parameters associated with the processes. The changes in the secondary and tertiary structural folds were measured by CD and intrinsic fluorescence-based spectroscopic methods. Unfolding transition behavior along with the parameters like melting temperature (T_m_), mid-point of the unfolding transition (D_m_) and the apparent standard free energy change parameters $\left( {\Delta {\text{G}}_{{{\text{NU}}}}^{{{\text{H}}_{2} {\text{O}}}} } \right)$ were further elucidated for the AAR protein. These data were indicative of the marginal stability of the AAR protein which is a plausible reason for the aggregation propensity, low turnover number and hence low catalytic activity of the enzyme when expressed in *E. coli*. Furthermore, molecular dynamics (MD) simulation conducted on AAR structure enabled us to understand the positive effect of ionic solvent KCl on protein structure stabilization.

## Results

### Expression, purification and functional characterization of AAR

For overexpressing AAR, the codon-optimized *aar* gene (from *S. elongatus* PCC 7942 strain) was cloned into the bacterial expression vector pQE30^8^, transformed into M15 strain of *E. coli*, and induced in liquid culture using 1 mM IPTG. The induced culture was shifted to 18 °C and cells were harvested after 16 h of post-induction. SDS-PAGE analysis of the induced culture pellet revealed an intense band around 38 kDa, which was consistent with the calculated molecular mass of AAR and was absent in the uninduced cells (Fig. 1B).

The 6-His tagged recombinant AAR was purified from the soluble fraction of the expressed protein obtained after cell lysis to ensure the isolation of natively folded protein. The purification of AAR was carried out in a two-step manner (Fig. 1B). The first step of purification involved Ni–NTA based affinity chromatography, where major contaminant proteins in the supernatant were removed. The employment of the second step, i.e. gel filtration chromatography, helped in the removal of the residual non-AAR proteins (Fig. S2) and the purified AAR fractions were pooled, concentrated and analyzed by SDS-PAGE (Fig. 1B). The results suggest that AAR has been successfully purified by the two steps of chromatographic purification.

**Figure 2:**
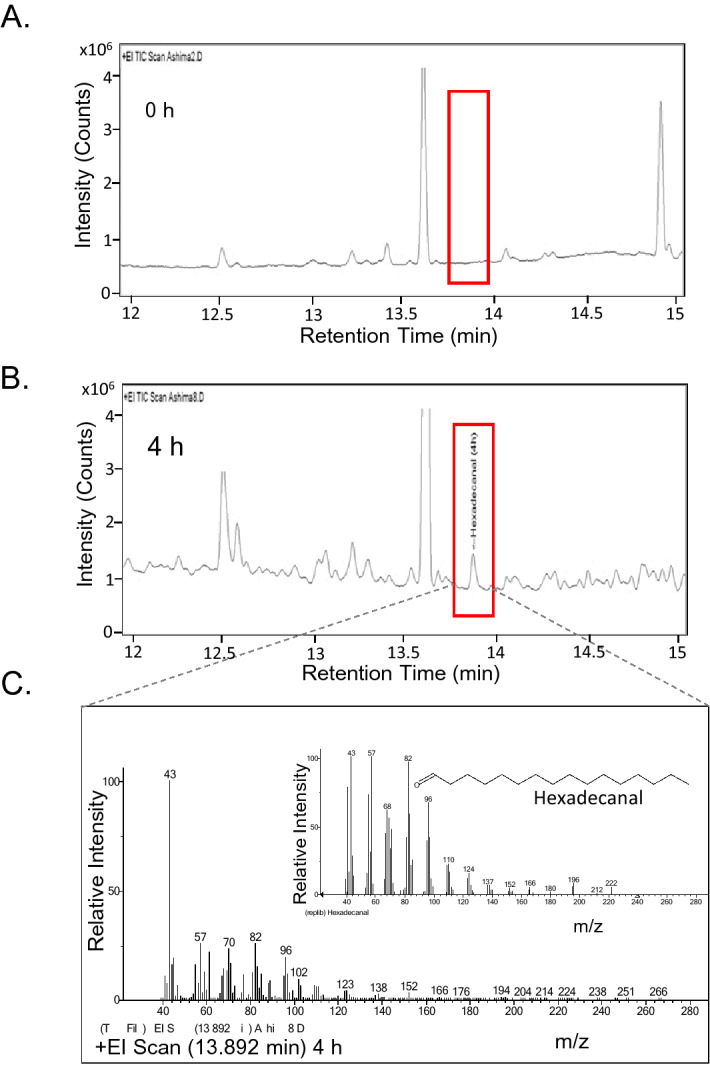
Enzymatic activity assay of AAR. Panels represent Total Ion Current (TIC) chromatogram probed by Gas Chromatography-Mass spectrometry (GC–MS) of the analyzed reaction product (hexadecanal) at different time intervals 0 h (**A**) and 4 h (**B**). (**C**) Mass spectrum of the peak corresponding to hexadecanal, Inset represents the mass spectrum of the hexadecanal from the database library.

Functional AAR is known to exhibit reductase activity catalyzing the acyl-CoA/ACP substrate to its corresponding aldehyde. We checked the activity of the purified AAR using palmitoyl-CoA and NADPH as substrates and analyzed the products of the reaction at different time intervals by GC–MS (Fig. 2). The maximum product concentration peak with retention time 13.9 min, corresponding to 4.7 µg/mL hexadecanal, was achieved at 4 h of incubation period (Fig. 2A, B), as analyzed by GC–MS profile (Fig. 2C). The result suggests that the purified AAR is a functionally active protein.

### Determination of disperse nature of purified AAR

**Figure 3:**
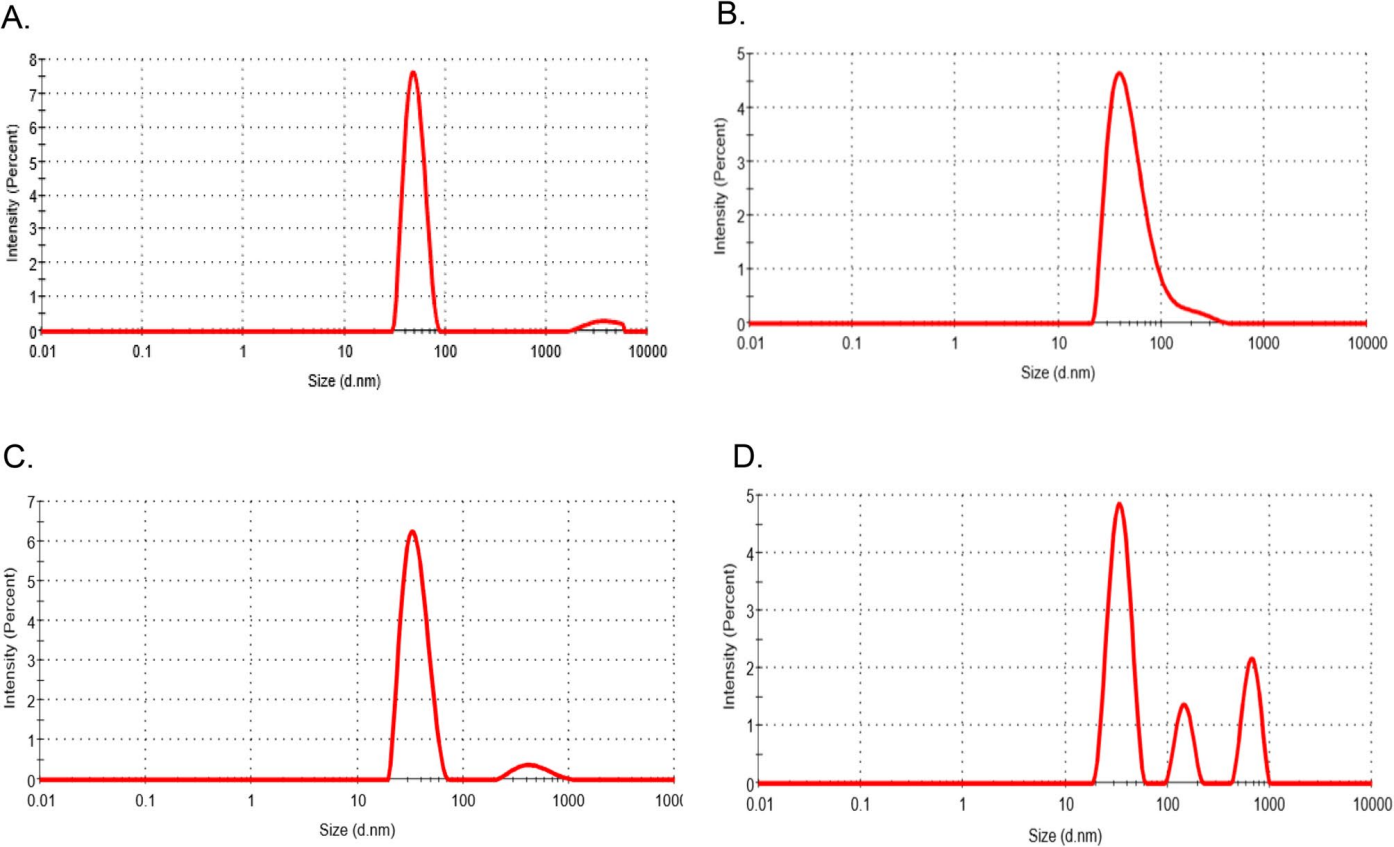
Dynamic-light-scattering distribution curves of AAR. Panel A, B, C, and D represent intensity size distribution curves for AAR GFC pooled fractions a, b, c, and d, respectively, obtained using Malvern Zetasizer software (version 7.12). The concentration of each pooled fraction was 1 mg/mL.

We realized from the GFC profile that AAR eluted at wide elution volume rather than a sharp peak (Fig. S2). To understand the nature of AAR eluted at different retention time, we determined its dispersity via dynamic light scattering (DLS) experiment. AAR GFC elutes were divided into four different fractions, namely a, b, c, d (Fig. S2), pooled and concentrated to ~ 1 mg/mL for performing DLS. It was observed that a, b, and c fractions were monodisperse with varying hydrodynamic diameter of 51.6 nm, 45.3 nm, 36.4 nm, respectively (Fig. 3). On the other hand, Fraction d was polydisperse in nature. Fraction a, b, and c corresponds to the molecular weight of AAR when analyzed on SDS-PAGE (Fig. S2) and exhibits enzymatic activity (Fig. 2). Together when pooled, fractions a, b, and c corresponds to a polydisperse state of solution (Fig. S3). It is indicative of multiple-sized protein particles that exist in AAR purified solution as depicted from the GFC profile of AAR as well (Fig. S2), thus corresponding to different states of AAR.

### Analysis of intact nature of AAR using LC–mass spectrometry

**Figure 4:**
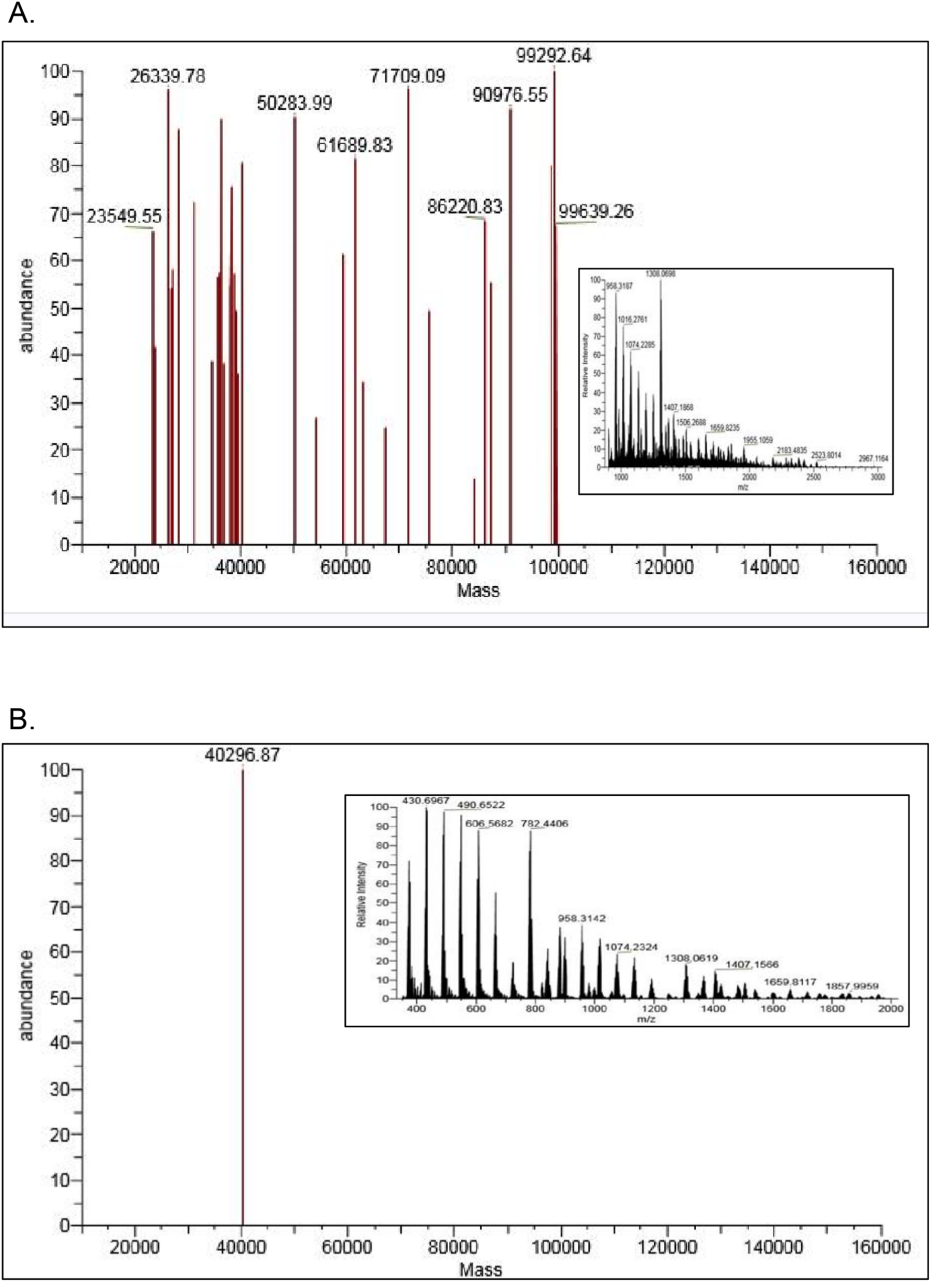
Intact mass determination of AAR using LC-ESI Mass spectroscopy. (**A**) Deconvoluted mass spectrum of AAR; inset shows full ESI–MS Scan profile of AAR. (**B**) Intact mass determination of de-lipidated AAR using LC-ESI Mass spectroscopy-deconvoluted Mass spectrum of MAYI-ODS treated AAR; inset shows full ESI–MS scan of MAYI-ODS treated AAR.

Native protein intact mass determination of purified AAR was carried out using ESI-mass spectrometry coupled to UHPLC for the upfront separation system. Figure 4A represents the deconvoluted mass spectrum of AAR derived from the full ESI–MS m/z scan (inset). Purified AAR appeared to adopt heterogeneous states in the solution with mass ranging from 20 to 100 kDa (Fig. 4A), which was in agreement with the DLS data (Fig. 3). Interestingly, when purified AAR was subjected to de-lipidation using Shimadzu MAYI-ODS column prior to LC-ESI MS analysis, it resulted in a single mass peak of 40,296.87 Da (Fig. 4B). The mass corresponded to the single monomeric molecular weight of AAR and no heterogeneous states could be observed in the deconvoluted spectra (Fig. 4B) as compared to the un-treated AAR (Fig. 4A). This result suggested that some lipid molecules bind to AAR during its heterologous expression in *E. coli*.

To further explore the nature of lipid molecule bound to AAR, we carried out the LC–MS analysis of the lipid extract from the Octadecyl-silica (ODS) column (Fig. S4). The characterization of the lipids binding to AAR protein was qualitative in nature by using a non-targeted profiling method. LC–MS fragmentation data was searched across the database LipidSearch (version 4.1.16, Thermo Fischer Scientific) and resulted in list of proposed lipid moieties binding to the AAR as shown in Table S1. The most prominent of these were fatty acids, hydroxy fatty acids, and prenol lipid.

### Determination of structural integrity of AAR

**Figure 5:**
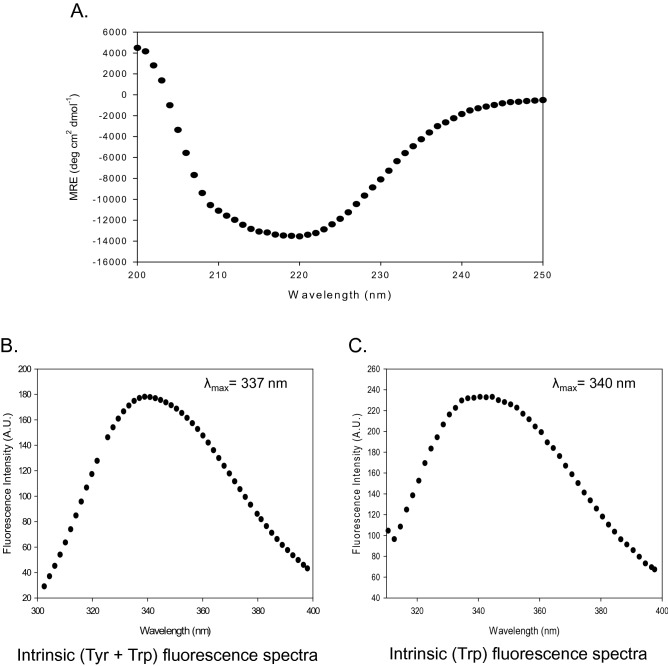
Secondary and tertiary structure determination of AAR. (**A**) Graph shows Far-UV CD spectra of AAR (3.5 µM) monitored from 250 to 200 nm wavelength to determine secondary structure. Tertiary structure determination of AAR probed by intrinsic fluorescence spectroscopy was performed by (**B**) Intrinsic (Trp + Tyr) emission fluorescence spectra of AAR (3.5 µM) with excitation at 280 nm, and (**C**) intrinsic (Trp) emission fluorescence spectra of AAR with excitation at 295 nm.

The secondary structure of AAR was determined by monitoring far-UV CD spectra from wavelength 200 to 250 nm as shown in Fig. 5A. Through examining DLS-derived size distribution by number (%) and volume (%), it was observed that the mean particle size of fraction b and c is comparable (Fig. S7) with similar CD and intrinsic fluorescence spectra (Fig. S9B, C). When pooled together, fraction b and c corresponded to a monodisperse solution state (Fig. S9A) thus these two fractions were pooled and considered for further analysis. The CD spectra were used for the calculation of the percentage of the helix, sheets, turns, and random structure for AAR protein. The percentage of helix, sheet, turns, and random structures were approximately 39.1%, 22.1%, 9.7%, and 28.1%, respectively, by using Yang’s reference software. The result was in agreement with the reported secondary structure composition of AAR^15^ indicating that the purified protein has retained its native secondary structural folds. Intrinsic emission fluorescence of the aromatic acid residues in a protein was exploited for determining nature of the tertiary structural folds of the AAR protein by fluorescence spectroscopy. AAR comprises six tyrosine and six tryptophan residues. The protein was excited at 280 nm and 295 nm to obtain intrinsic ‘Tyr + Trp’ and intrinsic ‘Trp’ fluorescence spectra, respectively (Fig. 5B, C). The emission maxima (λ_max_) of AAR when excited at 280 nm and 295 nm wavelengths were 337 and 340 nm, respectively (Fig. 5B, C). The λ_max_ values correspond to the emission of Trp residue when not exposed to the polar solvent environment, indicative of buried residues in a hydrophobic environment which is the characteristic of a folded protein. These results, therefore, suggested that purified AAR existed in its correctly folded native form.

### Thermal unfolding and refolding studies of AAR

The surrounding environment, to which a protein is being subjected to, plays a crucial role in dictating its folding and stability in the cellular condition. Exposure of protein to unfavorable conditions accounts for its misfolding or unfolding leading to aggregation. To understand the effect of one of the critical environmental variables like temperature on the structural integrity of the AAR protein, we have carried out thermal-induced unfolding and refolding studies.

#### Study of thermal unfolding and refolding of AAR by CD spectroscopy

**Figure 6:**
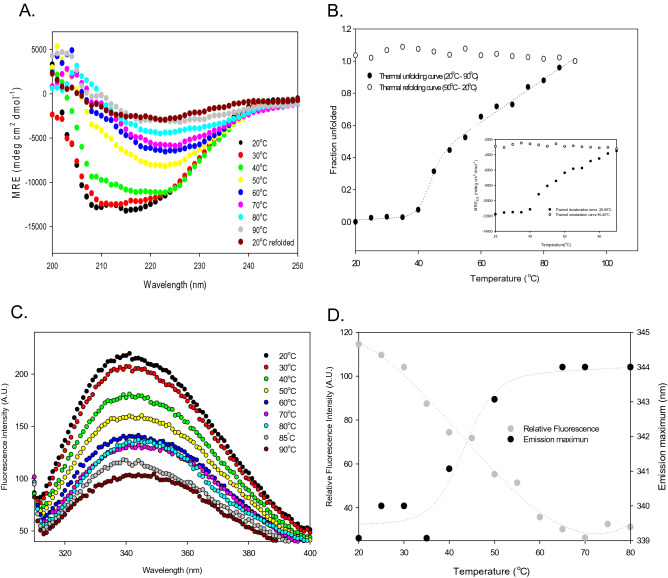
Thermal unfolding and refolding profile of AAR. Upper panel represents thermal unfolding and refolding probed by CD spectroscopy. (**A**) Graph shows CD spectra of AAR (3.5 µM) when subjected to different temperatures varying from 20 to 90 °C. (**B**) Graph represents thermal unfolding (20–90 °C) and refolding transition (90–20 °C) curve of AAR monitored by CD spectroscopy at 222 nm. The intercept and slope of baselines, of the unfolding transition curve, were used for fitting data in two state model represented by dotted line. The inset of graph represents the MRE_222_ values of AAR protein corresponding to different temperatures during thermal unfolding and refolding transition. Lower panel represents thermal unfolding and refolding probed by intrinsic fluorescence spectroscopy. (**C**) Graph represents unfolding of AAR subjected to different temperatures from 20 to 90 °C. Fluorescence emission spectra of AAR monitored from 300 to 400 nm when excited at 295 nm at varying temperatures. Fluorescence intensity decreases with increase in temperature. (**D**) Graph represents the relative fluorescence intensity at 340 nm of AAR subjected to different temperatures (20–80 °C). The secondary axis plot shows the change in the emission maximum (λ_max_) with increasing temperature. The intercept and slope of baselines, of the unfolding transition curve, were used for fitting of data in two state unfolding model.

Thermal unfolding and refolding of AAR was probed by CD spectroscopy. Figure 6A represents changes in the CD spectra of AAR when subjected to different temperatures corresponding to the changes in the secondary structure of the protein. The unfolding transition of AAR was monitored at 222 nm upon subjecting to temperature range varying from 20 to 90 °C (Fig. 6A). With AAR being subjected to an end denaturation temperature of 90 °C, the loss of secondary structure could be observed. A typical sigmoidal curve was obtained with increasing temperature indicative of almost complete unfolding of protein and loss of its secondary structure (Fig. 6B). The curve also suggested that the secondary structure of AAR remained intact up to 35 °C, with eventual conformational change occurring till 60 °C followed by the continuous loss of secondary structure at higher temperatures. The melting temperature of AAR was determined by fitting the thermal denaturation transition curve to a two-state model and was found to be 43.3 ± 0.9 °C (Table 1). The experimental data fitting coincided with the theoretical data fitting line for the two-state (Fig. 6B). The 90 °C heat-denatured protein when cooled gradually from 90 to 20 °C was unable to attain the folded native conformation (Fig. 6B). This shows the process is irreversible in nature as depicted.

**Table 1:** Melting temperature (T_m_) of AAR from thermal unfolding transition using different probes.

S. No	Probe	Melting temperature (T_m_) (°C)^a^
1	CD spectroscopy	43.3 ± 0.9
2	Intrinsic fluorescence spectroscopy	42.8 ± 2.1

^a^The values are mean of three measurements along with the standard error of the mean.

#### Thermal unfolding and refolding of AAR probed by intrinsic fluorescence spectroscopy

Changes in the tertiary structure of AAR with increasing temperature was also monitored by recording changes in intrinsic tryptophan fluorescence intensity with continuous scan from 20 to 90 °C (Fig. 6C, D). A continuous decrease in the fluorescence intensity could be observed with increasing temperature (Fig. 6C). The relative fluorescence intensity corresponding to the respective temperature was plotted. The tertiary structure of AAR was retained until 30 °C as no change in the emission maximum was observed (Fig. 6D). This was followed by a gradual red-shift in the λ_max_ from 340 to 344 nm up to 60 °C indicating the conformational changes in the protein and further leading to denaturation at higher temperatures (Fig. 6C). Similar to CD, fluorescence data was also fitted to a two-state model and fitting parameters obtained were useful in determination of the melting temperature of AAR to be 42.8 ± 2.1 °C (Table 1).

### Denaturant-mediated equilibrium unfolding of AAR

To comprehend the nature of interactions mainly responsible for stabilizing the AAR protein, effect of different solvent composition on the structural integrity of the protein was studied via chemical denaturant-mediated equilibrium unfolding studies. AAR was subjected to two different denaturants exhibiting different chemical characteristics, namely urea and GdnHCl, and the effect on the conformation and stability of the protein was studied.

#### Urea-mediated equilibrium unfolding of AAR probed by CD spectroscopy

**Figure 7:**
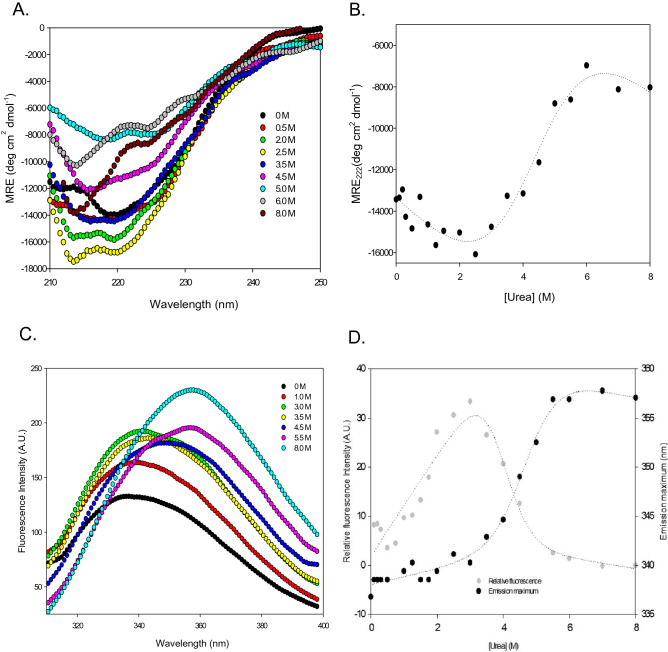
Urea-mediated equilibrium unfolding of AAR. Upper panel represents urea-mediated equilibrium unfolding probed by CD spectroscopy. (**A**) Graph shows far-UV CD spectra of AAR at different urea concentrations. Lower panel represents urea-mediated unfolding probed by intrinsic fluorescence spectroscopy. (**B**) Graph shows the urea-induced unfolding profile of AAR (3.5 µM) monitored by far-UV CD at 222 nm at different varying concentrations of urea. The intercept and slope of baselines, of the unfolding transition curve, were used for fitting of data in Eq. (6) (data analysis section). (**C**) Graph shows fluorescence emission spectra of AAR monitored from 310 to 400 nm when excited at 295 nm at different concentration of urea. (**D**) Graph represents the relative fluorescence intensity of AAR at 340 nm subjected to varying urea concentration. The secondary axis plot shows the change in the emission maximum (λ_max_) at different concentrations of urea. The intercept and slope of baselines, of the unfolding transition curve, were used for fitting of data in Eq. (6) (data analysis section).

The changes in ellipticity at 200–250 nm were measured at different urea concentrations from (0–8 M) (Figs. 7A and S8). The MRE (molar residue ellipticity) values calculated at 222 nm for each sample were plotted against the respective urea concentration from 0 to 8 M to obtain an unfolding transition curve (Fig. 7B). It was observed that the secondary structure of the protein remained similar when concentration of urea was increased from 0 to 0.75 M (Fig. S8B). This was followed by increased negative CD signal of AAR observed till 2.5 M concentration of urea (Fig. S8B). Beyond 2.5 M concentration of urea, a decrease in the value of negative MRE suggests a continuous loss of secondary structure, and onset of unfolding transition, till 6.0 M urea concentration. Eventually, at 6.0 M urea concentration, the maximum unfolded fraction of protein was obtained which indicated a complete loss of the secondary structure of the protein (Fig. 7A,B). The unfolding transition curve was fitted into the two-state transition model (Eq. 6), and the D_m_ value (denaturant concentration at which 50% of the protein is unfolded) was calculated to be 4.3 ± 0.4 M. The apparent standard free energy change was also calculated and found to be 4.3 ± 1.0 kcal mol^−1^ respectively (Table 2). The urea-induced unfolding of AAR can be represented by the following scheme:

$${\text{Native}}\;\left( {\text{N}} \right) \to {\text{Unfolded}}\;{\text{state}}\;\left( {\text{U}} \right)$$

**Table 2:** Urea-induced equilibrium unfolding parameters of AAR.

S. No	Probe	D_m_ (M)	m_NU_ (kcal/mol M)	Apparent standard free energy change (kcal mol^−1^)
1	CD spectroscopy	4.3 ± 0.4	0.8 ± 0.24	4.32 ± 1.0
2	Intrinsic fluorescence	3.9 ± 0.2	1.5 ± 0.63	5.45 ± 0.9

The values are mean of three measurements along with the standard error of the mean.

#### Urea-mediated equilibrium unfolding of AAR probed by intrinsic fluorescence spectroscopy

The change in fluorescence emission intensity at 340 nm and λ_max_ in the presence of increasing concentration of urea for AAR protein have been shown in Fig. 7C, D. It was observed that the fluorescence intensity of AAR was higher in the presence of lower concentrations of urea which further increased to the maximum value at 2.5 M with unaltered λ_max_ (340 nm) of emission (Fig. S8B). These initial changes in fluorescence intensity without affecting λ_max_ are readily observed at low urea concentrations and could be a result of environmental alteration around the quenched tryptophan residues in the fully folded protein. The fluorescence intensity started decreasing beyond 2.5 M with the gradual redshift in the λ_max_ representing the onset of the structure unfolding (Fig. 7C). A significant decrement in the fluorescence intensity and an appreciable redshift in the λ_max_ (357 nm) were observed at 5.5 M with no further apparent changes (Fig. 7D), representing the exposure of aromatic amino acid residues to the environment and complete loss of AAR tertiary structure. The unfolding transition curve was fitted into the two-state transition model (Eq. 6) (Fig. 7D). The D_m_ value from the model was calculated to be 4.7 ± 0.2 M and the apparent standard free energy change was calculated to be 5.4 ± 0.9 kcal mol^−1^ (Table 2). The results obtained here further validate the earlier-calculated parameters from the CD data.

#### GdnHCl-mediated equilibrium unfolding of AAR probed by CD spectroscopy

**Figure 8:**
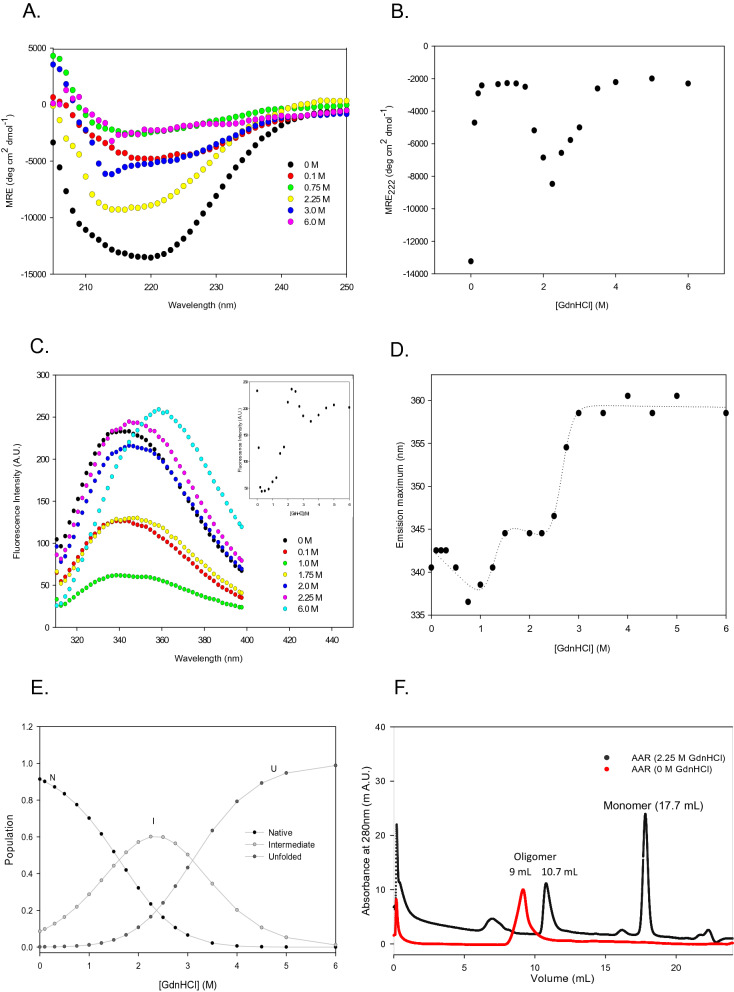
GdnHCl-mediated equilibrium unfolding of AAR. Upper panel represents GdnHCl-mediated equilibrium unfolding of AAR probed by CD spectroscopy. (**A**) Graph shows far-UV CD spectra of AAR at different GdnHCl concentrations. (**B**) Graph shows the GdnHCl-induced unfolding profile of AAR (3.5 µM) monitored by far-UV CD at 222 nm at different varying concentration of denaturant. The intercept and slope of baselines, of the unfolding transition curve, were used for fitting of data in Eq. (11) (data analysis section). Middle panel represents GdnHCl-mediated equilibrium unfolding of AAR probed by intrinsic fluorescence spectroscopy. (**C**) Graph shows fluorescence emission spectra of AAR monitored from 310 to 400 nm when excited at 295 nm at different concentration of GdnHCl. The inset graph represents the fluorescence intensity of AAR at 340 nm when subjected to varying AAR concentration. (**D**) Graph shows the change in the emission maximum (λ_max_) at different concentration of urea. The intercept and slope of baselines, of the unfolding transition curve, were used for fitting of data in Eq. (11) (data analysis section). (**E**) Panel represents theoretical curve showing different population during GdnHCl-induced unfolding pathway of AAR. Graph shows the GdnHCl concentration dependence of populations of native (N), intermediate (I) and unfolded state (U) of AAR calculated from the thermodynamic parameters listed in Table 3. (**F**) Graph shows size exclusion chromatography elution profile of AAR at 0 M and AAR at 2.25 M concentration of GdnHCl using superdex 200 increase 10/300 GL column.

The far-UV CD spectra of AAR in the presence of different GdnHCl concentrations have been presented in Figs. 8A and S6a. A three-step change of CD signal at 222 nm was observed with increasing concentrations of GdnHCl (Fig. 8B). The first transition occurred from 0 and 1.25 M GdnHCl and was followed by an increase of CD signal from 1.25 to 2.25 M and complete unfolding occurred over 3.0 M GdnHCl (Fig. 8B). The CD signal at 222 nm was lost during the first transition, indicating partial unfolding of AAR molecules at lower denaturant concentrations. However, the CD signal at 222 nm increased again during the second transition with a further increase of GdnHCl concentration, regaining approximately 60% of the native secondary structure. Then, a complete loss of secondary structure occurred during the third transition, showing that AAR adopted a complete unfolded state. The result obtained indicates that the AAR had undergone GdnHCl-mediated complete unfolding via intermediate formation thus following the 3-state folding transition. The unfolding transition curve was fitted into the three-state protein folding model (Eq. 11). The D_m_ (NI), Dm (NU) were determined to be 1.7 ± 0.05 M and 3.1 ± 0.3 M respectively (Table 3). Apparent standard free energy of unfolding of the transitions from native to intermediate ${\text{dG}}_{{{\text{NI}}}}^{{{\text{H}}_{2} {\text{O}}}}$, intermediate to unfolded ${\text{dG}}_{{{\text{IU}}}}^{{{\text{H}}_{2} {\text{O}}}}$, and native to unfolded ${\text{dG}}_{{{\text{NU}}}}^{{{\text{H}}_{2} {\text{O}}}}$ state was determined to be 1.5 ± 0.1 kcal mol^−1^, 2.8 ± 0.5 kcal mol^−1^, and 4.3 ± 0.5 kcal mol^−1^, respectively (Table 3).Therefore, GdnHCl-induced unfolding can be represented by the following scheme:

$${\text{Native}}\;\left( {\text{N}} \right) \to {\text{Intermediate}}\;{\text{state }}\left( {\text{I}} \right) \to {\text{Unfolded}}\;{\text{state}}\;\left( {\text{U}} \right)$$

**Table 3:** GdnHCl-mediated equilibrium unfolding parameters of AAR.

Probe	D_m NI_ (M)	D_m NU_ (M)	${\text{dG}}_{{{\text{NI}}}}^{{{\text{H}}_{2} {\text{O}}}}$(kcal/mol)	${\text{dG}}_{{{\text{NU}}}}^{{{\text{H}}_{2} {\text{O}}}}$(kcal/mol)	${\text{dG}}_{{{\text{IU}}}}^{{{\text{H}}_{2} {\text{O}}}}$(kcal/mol)	m_NI_ (kcal/mol M)	m_NU_ (kcal/mol M)	m_IU_ (kcal/mol M)
CD	1.7 ± 0.05	3.1 ± 0.3	1.5 ± 0.1	4.3 ± 0.5	2.8 ± 0.5	0.9 ± 0.1	1.4 ± 0.3	0.5 ± 0.3
Fluorescence	1.6 ± 0.2	2.6 ± 0.1	1.4 ± 0.1	4.7 ± 0.5	3.3 ± 0.4	0.8 ± 0.7	1.7 ± 0.5	0.9 ± 0.2

The values are mean of three measurements along with the standard error of the mean.

#### GdnHCl-mediated equilibrium unfolding of AAR probed by intrinsic fluorescence spectroscopy

The AAR unfolding transition was monitored at different GdnHCl concentrations through the changes of fluorescence intensity and the emission maximum wavelength (λ_max_). Figures 8C and S6b showed the fluorescence spectra and fluorescence intensity of AAR at different GdnHCl concentration, and Fig. 8D showed the changes in the λ_max_ of AAR in the presence of increasing GdnHCl concentrations. We observed the initial decrease in the fluorescence intensity and λ_max_ at a low concentration of the denaturant, indicating altered protein native structure. The unfolding induced by GdnHCl exhibited two transition processes. During the first transition, an increase in fluorescence intensity and a redshift in the λ_max_ was observed with increasing GdnHCl concentration from 1.0 to 2.25 M. This was indicative of intermediate formation which had a fluorescence quantum yield similar to the native AAR. The fluorescence spectrum of this intermediate was slightly red-shifted, suggesting that the Trp residues’ environments were not appreciably altered. A second transition was represented by a clear decline in the intensity and notable red-shift in the λ_max_, 345 nm to 360 nm, upon increasing GdnHCl concentration from 2.25 to 3.0 M with no apparent changes further. This redshift in λ_max_ showed Trp residues exposure to a polar environment as a result of complete unfolding of AAR at higher GdnHCl concentration. The unfolding transition curve was fitted into the three-state transition model (Eq. 11), and the D_m_ (NI) and Dm (NU) were determined to be 1.6 ± 0.2 M and 2.6 ± 0.1 M, respectively (Table 3). Apparent standard free energy of unfolding of the transitions from native to intermediate ${\text{dG}}_{{{\text{NI}}}}^{{{\text{H}}_{2} {\text{O}}}}$, intermediate to unfolded ${\text{dG}}_{{{\text{IU}}}}^{{{\text{H}}_{2} {\text{O}}}}$, and native to unfolded ${\text{dG}}_{{{\text{NU}}}}^{{{\text{H}}_{2} {\text{O}}}}$ state was determined to be 1.4 ± 0.1 kcal mol^−1^, 3.3 ± 0.4 kcal mol^−1^, and 4.7 ± 0.5 kcal mol^−1^, respectively (Table 3). The results obtained were in agreement with the CD data showing that AAR follows a 3-state GdnHCl –mediated unfolding transition via intermediate formation. From the theoretical curves, the apparent fraction of native (f_N_), intermediate (f_I_ ) and unfolded (f_U_) protein with respect to denaturant concentration were calculated by using Eq. 9 (Fig. 8E). The maximum fractional population (~ 60%) of intermediate state for the protein was observed at 2.25 M GdnHCl concentration.

### Computational evaluation of AAR stability and interactions in presence of KCl

KCl, an ionic solvent with similar properties as GdnHCl at lower concentrations, has been reported to enhance AAR activity significantly^11,15^ After an extensive biophysical characterization of AAR exhibiting different unfolding properties in presence of different solvent environments, we wanted to understand the effect of KCl using computational modelling and simulation methods. As presence of an intermediate of AAR at lower concentration of ionic solvent has been observed in the present study, we were inquisitive to understand the effect of KCl on structure and stability of AAR.

**Figure 9:**
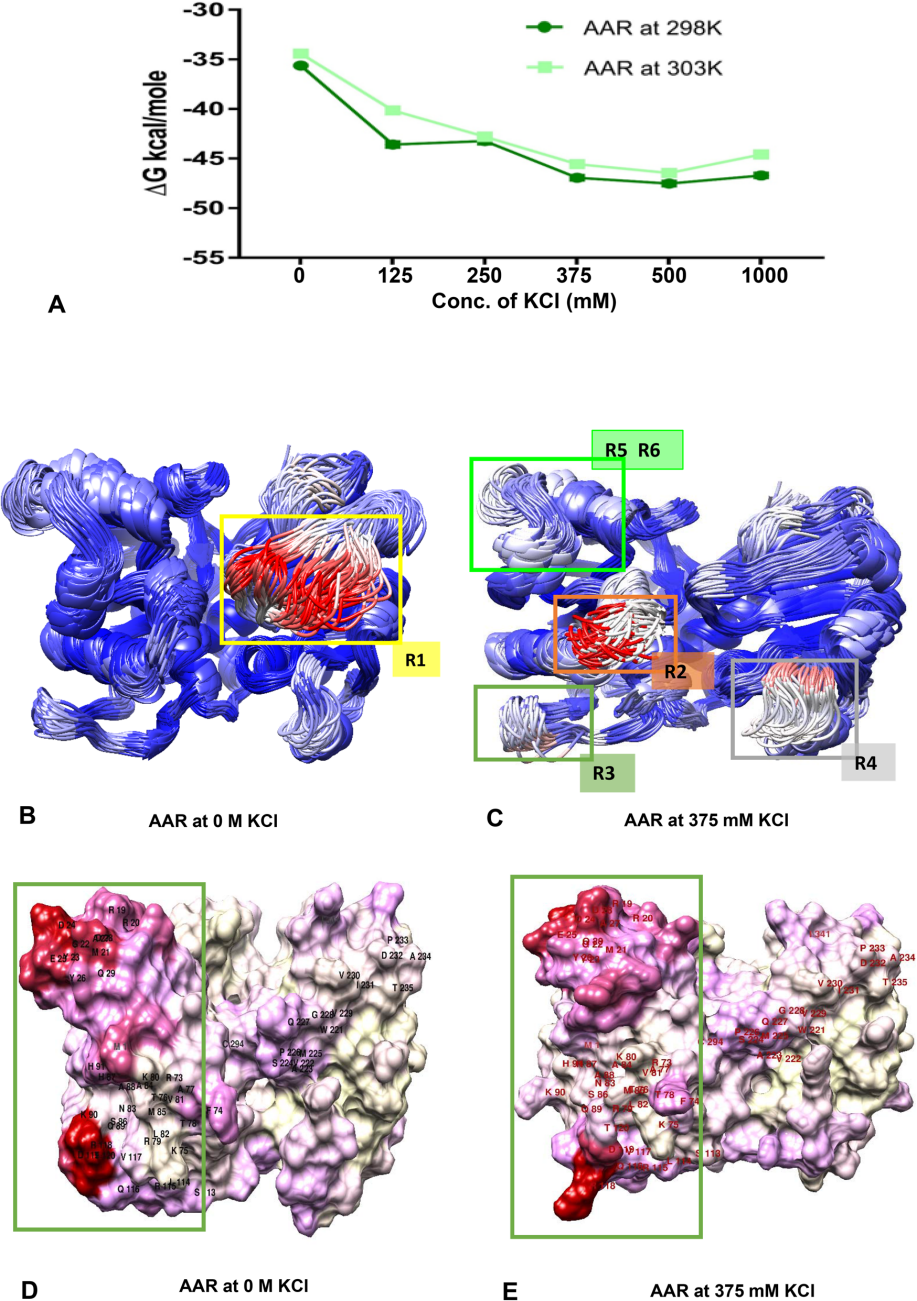
Molecular dynamics simulations of AAR. (**A**) The free energy values of AAR subjected to different concentrations of KCl ranging from 0 to 1000 mM. Energies were derived from Molecular Mechanics Generalized Born Surface Area (MMGBSA) calculations. (**B**, **C**) The cluster representations of AAR at 0 M and 375 mM KCl over a 200-ns trajectory at 2-ns intervals. The enzymes are colored by RMSF, where red represents the highest fluctuations, and blue represents the lowest fluctuations. Highlighted R1, R2, R3, R4, R5, and R6 correspond to (A223-V230), (L69-R72), (L114-E123), (A188-N190), (A14-D16), and (D28-S35) regions of AAR respectively. (**D**, **E**). The surface view of the cluster representative of AAR subjected to 0 M and 375 mM KCl solvent environment, here red and cream color represent maximum and minimum difference respectively in their structures when aligned and compared with each other. Chimera tool^32^ was used for visualizing these structures.

Molecular dynamics simulations of AAR (PDB ID: 6JZU) in solution with varying concentrations of KCl were carried out. Simulations of the enzyme in each representative state enabled us to directly examine the effects of KCl on protein dynamics. The energy decomposition of the AAR under different simulation environment (0–1000 mM KCl) at two different temperatures 298 K and 303 K is shown in Fig. 9A. AAR with increasing concentration of KCl seemed to attain more energetically favored state, especially at 375 mM conc. of KCl. For each simulation, the root-mean square deviations (RMSD) with reference to initial structure used in simulations of the protein were calculated (Fig. S5A). Our results show that the proteins were mostly stable and reached geometrical convergence after 500 ns timescale at different applied conditions.

Afterward, to identify the regions of the protein exhibiting differences in the fluctuations, RMSD of the protein (backbone) was calculated for the protein by extracting 100 snapshots at 2 ns intervals from the last 200 ns of the whole MD trajectories and then aligning each one of them with the first frame of the extracted frames at different concentrations of KCl (0 and 375 mM of KCl) (Fig. S5b). The enzymes were colored on the basis of the level of fluctuations observed, where blue and red represent the lowest and highest fluctuations, respectively, at 0 M and 375 mM of KCl (Fig. 9B, C). Overall shape of the AAR protein was observed to be compactly packed in presence of 375 mM of KCl. Additionally, comparing the regions of highest fluctuations of AAR protein at 0 M and 375 mM of KCl, we observed differences primarily in the N-terminal domain (R2, R3, R5, and R6) and mid domain regions of the protein (R1, R4) as represented in Fig. 9B, C.

Residues in the regions R2 (L69-R72), R3 (L114-E123), R5 (A14-D16), and R6 (D28-S35) demonstrated increased fluctuation when AAR was exposed to 375 mM of KCl. All these regions belonged to N-terminal domain of AAR where the binding of next enzyme in the pathway, i.e., ADO, was observed earlier^15^. Residues in the region R1 (A223-V230) on the other hand exhibited higher fluctuation in absence of KCl. R1 region belongs to the intrinsic flexible region III of the mid domain of AAR and in the presence of 375 mM of KCl diminished RMSF values were observed.

The surface view of the cluster representative of AAR under subjected conditions (Fig. 9D,E) clearly represented the differences in the structure of AAR as represented by the marked regions in the presence and absence of KCl in the solvent environment. Interestingly, a more compact overall structure with major structural changes in the N-terminal regions of AAR associated with binding to ADO protein was observed in presence of KCl salt as compared to the control.

## Discussion

Schirmer group in 2010 had identified two enzymes of the alkane biosynthetic enzyme pathway^10^. Since then, extensive structural characterization of the terminal ADO protein and its variants had been carried out^2^. Surprisingly for AAR, the structural aspects still remain largely obscure generating a wide gap in the literature. The matter becomes crucial to be investigated considering the colossal biotechnological relevance of the protein and diminishingly existing structural information.

In the present communication, we have attempted to isolate and carry out a detailed characterization of the AAR protein. The AAR protein from *Synechococcus elongatus* PCC7942 had been reported to exhibit the maximum activity among other cyanobacterial AARs^14^. We studied the expression of this protein in *E. coli* since the maximum achieved hydrocarbon production to date has been reported in this host^8^. However, AAR, a water-soluble cyanobacterial enzyme, unlike ADO is prone to aggregation when expressed recombinantly in the heterologous host. The present work has successfully attempted to investigate the reasons associated with the structural discrepancies exhibited by expressed AAR and shortlisted the probable bottlenecks associated with its aggregation which could be targeted and resolved for achieving improved hydrocarbon production.

AAR protein has been expressed and successfully purified from the *E. coli* host (Fig. 1). The enzyme has exhibited reductase activity thus confirming that the purified AAR is a functionally active protein (Fig. 2). Interestingly, GFC elution profile of AAR does not correspond to a monomeric fraction, instead, a broadly distributed peak-like pattern has been observed indicative of the co-existence of multiple-sized states of the protein in solution (Fig. S2), which was also previously reported by Kudo et al. group^17^. The presence of other protein contaminants was ruled out as only a single band was observed in SDS-PAGE corresponding to the monomeric molecular weight of the AAR protein (Figs. 1 and S2). The polydispersity of the AAR purified fraction was further established by DLS confirming with varied-sized protein particles in the solution (Fig. 3). Although it is difficult to quantify the oligomeric state, the data altogether offers an indication of size spread and relative contributions.

Additionally, mass spectrometry-based intact mass analysis of purified AAR exhibited irregularly distributed multiple states of AAR (Fig. 4A), further confirming the aforementioned results of GFC and DLS data. Excitingly, when the AAR purified fraction was subjected to de-lipidation, a single monomeric state of the protein dominated in the solution (Fig. 4B). The data acquired is intriguing as it suggests fatty acid-binding induces heterogenous multiple-sized states of the AAR protein. Reports are available where fatty acid-binding to another protein induces the oligomerization which can lead to aggregation of the protein^18^. Our results are also in concordance with the recent study by Gao et al.^15^ reporting that the AAR bound to its ligand could not generate stable crystals whereas apo-AAR could crystallize^15^. This inability seems to be the result of the ligand-induced heterogeneous population of AAR, which disappears once bound ligands (fatty acids) are removed, as evident from the current findings. Therefore, we propose that this could be one of the contributing reasons for enhanced aggregation propensity of the otherwise water-soluble AAR protein^9^ when expressed in the *E. coli* cells for hydrocarbon production. The current study accommodates comprehensive in vitro biophysical characterization of AAR to gain insights into the structural and stability aspects of the protein. It is possible that protein stabilities could be different inside cells than in vitro due to protein–protein interaction and other factors. However, few key experimental studies show that protein stability in cells is approximately the same as deduced in vitro^19–21^.

We have used far-UV CD, and intrinsic fluorescence spectroscopy to know about the secondary and tertiary structural folds of AAR protein. The calculated % of secondary structure from far-UV CD spectra were ~ 40% helix, ~ 22% sheets, ~ 10% turns, and ~ 28% random coil, which suggested a properly folded secondary structure (Fig. 5) and was in concordance with the recently published structure of AAR^15^. The emission fluorescence spectra of native AAR showed an emission maximum of 340 nm (Fig. 5). The λ_max_ values obtained (340 nm) corresponded to the emission of six Trp residues and was indicative of slightly exposed buried residues in a hydrophobic environment when not entirely exposed to the polar solvent, which is the characteristic of a folded protein. The results are therefore suggestive of purified AAR to exist in its folded native form.

Thermal stability is a critical property for many biotechnological applications of proteins as it implies longer life-times and frequently higher tolerance to the presence of organic co-solvents, extreme pH values, and high salt concentration or pressures^22^. In the present work, we have reported temperature-induced denaturation and renaturation studies of AAR.

AAR upon thermal denaturation, measured by CD and intrinsic fluoresce spectroscopy (Fig. 6), apparently displays a single transition. The protein resists change in its tertiary and secondary native structure till 30 °C and 35 °C respectively, followed by continuing structural transition and complete denaturation beyond 60 °C. The thermal denaturation process of AAR appears to be irreversible in nature (Fig. 6B). Melting temperature (T_m_, the temperature at which 50% of the protein is unfolded) of AAR was determined to be 43.3 ± 0.9 °C and 42.8 ± 2.1 °C by CD and intrinsic fluorescence spectroscopy, respectively. The thermal unfolding and refolding transition studies signify that the AAR fairly resists the change in structure upon unfolding as indicated by the thermal denaturation transition curve. But once the protein is denatured, it fails to adopt the native structure with a gradual lowering of the temperature thereby leading to irreversible denaturation and inactivation of the protein as demonstrated by the present study.

Interestingly, from this data, it could be inferred that the temperature range up to which AAR resists the change in its native structure is below the *E. coli* cultivation temperature at which the protein is usually expressed (30–37 °C) undergoing thermal stress and forms inclusion bodies. However, protein expression at lower temperature 18 °C improves its solubility^11^. This is suggestive that thermal stress is one of the contributing factors to the aggregation propensity of the protein when expressed in *E. coli* host. Loss of functional protein occurs due to irreversible degradation or misfolding or denaturation, of the otherwise water-soluble cyanobacterial enzyme, leading to aggregation in the heterologous host. Our results, therefore, indicate that there exists an immense scope to improve the thermal stability of the AAR protein so that the protein can withstand thermal stress when expressed in *E. coli* and end up not forming inclusion bodies.

The chemical stability of AAR protein has also been characterized by carrying out denaturant-mediated equilibrium unfolding studies. The unfolding processes of AAR induced by urea and guanidine hydrochloride (GdnHCl) were investigated by spectroscopic methods. In the unfolding processes, AAR tertiary structural transition was monitored by the changes of intrinsic fluorescence emission spectra, and its secondary structural transition was measured by the changes of far-UV CD spectra.

The urea-mediated unfolding of AAR appears to follow a single unfolding transition (Fig. 7). An increase in secondary structure content without any noticeable change in wavelength of emission maximum was observed till 2.5 M urea concentration, followed by a gradual loss in the structure of the protein till 6.0 M urea concentration beyond which no further loss in the structure has been observed. The redshift in the emission maximum accompanying the loss of secondary structure indicates protein to attain an unfolded state. AAR undergoes a co-operative two-state unfolding transition between N (native) and U (unfolded) state when treated with urea. The D_m_ (denaturant concentration at which 50% of the protein unfolded) and apparent standard free energy was determined to be ~ 4.0 M and ~ 5.0 kcal mol^−1^ respectively (Table 3).

At lower concentration of urea the protein conformation is influenced by an increase in the solvent structure and dielectric constant, which enhances the hydrophobic forces and thus results in increased ɑ-helical structure^23^. In the present data of urea-mediated unfolding of AAR, we have observed the same phenomenon of stabilization of protein structure at lower urea concentration reflected upon by a slight increase (~ 7%) in helical content of the protein when concentration of urea increased from 0 to 2.5 M (Fig. S8B). Additionally, fluorescence emission spectra at of ANS bound to AAR at 2.5 M and 0 M concentration of urea were also found to be similar (Fig. S8C).

The GdnHCl-induced equilibrium unfolding of AAR appears to follow the double sigmoidal curve indicative of two transitions, one at lower GdnHCl concentration while another at a concentration of 2.25 M GdnHCl (Fig. 8). This was followed by onset of complete unfolding of the protein beyond 3.0 M GdnHCl concentration. Due to the presence of more than one kind of observed population in the process, the data were fitted to a three-state model which explains the presence of N (native), I (intermediate), and U (unfolded) populations in the unfolding process (Fig. 8). Native protein population exists below 2.25 M GdnHCl concentration, the intermediate population is largely at the concentration of 2.25 M GdnHCl. From this data, it is reasonable to assume the existence of a three-state mechanism in which an intermediate is populated during the equilibrium unfolding process at 2.25 M concentration of GdnHCl (Fig. 8E). The percentage of intermediate state at 2.25 M GdnHCl concentration is about 60% (Fig. 8E).

Although native AAR showed partial unfolding at initial GdnHCl concentration, the intermediate state formed at 2.25 M concentration seems to regain nearly native-like secondary structure (CD spectra) (Fig. 8A), similar fluorescence quantum yield and similar fluorescence emission maxima (fluorescence spectra) (Fig. 8C). This could be attributed to the mode of the unfolding mechanism of GdnHCl considering that GdnHCl is a monovalent salt having both ionic and chaotropic effects^24,25^.

Guanidine is a salt expected to exist in the fully ionized form in aqueous solutions. The presence of Gdn^+^ and Cl^−^ influence the overall stability of proteins^24^. The stability of the intermediate is interpreted in terms of stabilization by generated Gdn^+^ and Cl^−^ ions masking the charged moieties of protein thereby reducing or even eliminating any destabilizing electrostatic interactions which has been reported for other enzymes as well^25,26^. Interestingly, AAR has been reported to interact with ADO primarily via electrostatic interactions^15^, thus retaining the exposed charged residues on its surface, which in the present case are stabilized at the lower concentration of the GdnHCl resulting in stabilization of the intermediate state of the protein. Strikingly, it is well established that the presence of KCl salt augments the AAR activity in a significant manner^11,15^. KCl is known to exhibit ionic character similar to the low concentration of GdnHCl^27^. The MD simulation studies revealed the effect of KCl on the N-terminal domain of AAR, responsible for interaction with the ADO protein, and also the diminished fluctuations in the flexible region III was observed when protein was subjected to solvent with 375 mM KCl (Fig. 9). The acidic residues (E196-E211) of this helix 7 of ADO are known to form strong electrostatic interactions with the basic residues from the long helical region (R73–H91), as well as R118 from flexible region II of AAR^15^. Interestingly a very significant difference in the surface properties of the AAR could be observed in these regions (Fig. 9C) in presence of KCl. The presence of KCl also energetically favored compact AAR structure as compared to the control (0 M) (Fig. 9A). Based on the present data it can be fairly explained that KCl stabilizes the AAR structure by masking the exposed electrostatic charge on the protein. The implications can be drawn that KCl-mediated improved activity of AAR is imparted through structural stabilization as reported previously for other enzymes as well^28^.

However, at higher concentration of GdnHCl acts as a classical denaturant leading to the unfolding of AAR protein chain as could be observed beyond 3.0 M concentration of GdnHCl (Fig. 8).

From the data, it could be extracted that though protein appears to resist change in the structure during unfolding till moderate concentration of denaturant interestingly the cooperativeness of the unfolding process is low as indicated by the cooperativity parameter (m) calculated by fitting the data in protein folding equation (Table 3). The low value of m indicates that the unfolding process follows multiple transitions rather than a single cooperative transition^29^ which contributes to the marginal stability of the protein. This low cooperativity in unfolding could be attributed to the recently reported intrinsic flexible regions in the AAR protein^15^.

AAR undergoes different modes of unfolding transition induced by chaotropic effects of urea and chaotropic as well as ionic effects of GdnHCl. The unfolding of AAR suggests different unfolding pathways and mechanisms for the two denaturants. The protein seems to use different pathways for unfolding in different milieu and is a classic example of how the environment dictates the path a protein might take to fold. The knowledge accumulated could be of immense biotechnological significance as well.

In the present study we have attempted to propose a tentative AAR unfolding scheme. AAR appears to exist in the native oligomeric state (Fig. 8F). Different unfolding schemes of AAR were observed in presence of different denaturants which can be comprehended as follows. It was observed that the AAR oligomer first dissociated to a folded monomer at low GdnHCl concentrations, and subsequently unfolded completely at higher GdnHCl concentrations (Fig. 8). There is no evidence for equilibrium intermediates, such as folded monomers, during AAR equilibrium denaturation induced by urea (Fig. 7). It can be elucidated that GdnHCl promotes oligomer dissociation to monomeric units followed by polypeptide unfolding, whereas urea promotes oligomer denaturation without disassembly.

GdnHCl-mediated unfolding process comprises of three transitions (Fig. 8). First unfolding transition (0–1.25 M) could be attributed to the oligomer dissociation as change in secondary structure (Fig. 8A) was observed with unaltered emission maximum wavelength and lower fluorescence intensity (Fig. 8C, D), indicative of the internal subunit conformational changes taking place within the oligomer assembly of AAR. This was followed by second transition (1.25 M to 2.25 M) where regaining of nearly native-like secondary structure (CD spectra) (Fig. 8A), similar fluorescence quantum yield and similar fluorescence emission maxima (Fluorescence spectra) (Fig. 8C) were observed, demonstrating populated AAR monomers dissociated from the oligomer. This was confirmed by the carried out SEC of AAR native and AAR incubated at 2.25 M concentration of GdnHCl (Fig. 8F). The monomer AAR state could be observed to populate at 2.25 M concentration of GdnHCl (Fig. 8F). The third transition is representative of AAR monomer unfolding (beyond 3.0 M GdnHCl), indicated by loss of secondary structure with red-shifted emission maximum wavelength corresponding to the lost tertiary structure.

The denaturant potential of GdnHCl has been attributed to general salt effects i.e., change in ionic strength on the polypeptide. Through the current findings, it appears that the dominant interaction of two interacting subunits in the native AAR oligomer involves charged residues. We speculate that interactions involving the charged residues are affected differently by a nonionic and an ionic denaturant so that the majority of subunit–subunit interactions can remain intact in the presence of high concentrations of urea, but not in the presence of high GdnHCl concentrations.

Interestingly, the denatured urea (8 M) and GdnHCl (6.0 M) state of AAR protein showed differences in the secondary structural content (Figs. 7A and 8A). The unfolding is more pronounced in case of GdnHCl-mediated process as compared to the urea mediated unfolding, again indicative of the fact that urea-mediated unfolding denaturation did not proceed via monomer formation. It appears that the finally urea–denatured state retains more secondary structural content as compared to that of GdnHCl-unfolded state. This could be indicative of retaining some structural content of the oligomer subunit held by electrostatic interactions which were disrupted in case of GdnHCl-mediated unfolding process which proceeds via oligomer dissociation.

The following scheme represents the two possible unfolding pathways in GdnHCl and urea solutions for AAR.
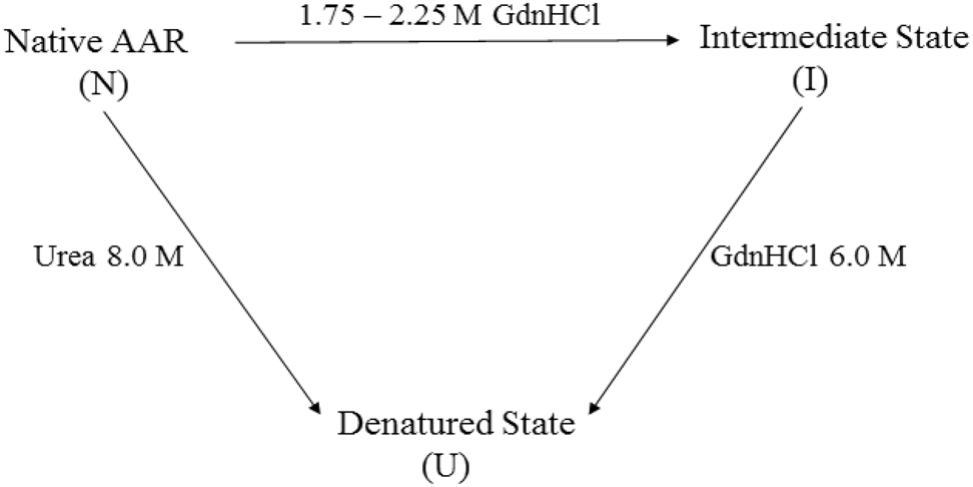


However, the apparent standard free energy deduced from the two processes is similar for AAR and is indicative of the marginal stability of the AAR protein, which is a plausible reason for the low turnover number and hence the low catalytic activity of the enzyme when expressed in *E. coli*. The present thermodynamic stability studies carried out are quite consistent with the recently published literature where AAR is reported to adopt lose conformation and is composed of three intrinsic flexible regions existing across its N-terminal domain and mid-domain^15^ which could be proposed to be contributing to the borderline stability of the enzyme. The work carried out in the present communication thus indicates that there exists a wide scope in improving the stability of this industrially relevant protein which in turn can improve the performance of the enzyme and the hydrocarbon production in the *E. coli* host.

In the *E. coli* proteome, it had been found that approximately 15% of proteins have a free energy change of ≤ 4.0 kcal/mol, while it undergoes an abrupt decrease once the cell is in thermal stress^19^. Hence, the above-calculated values of thermodynamic stability parameters for AAR reflect that it is a marginally stable entity (Tables 2 and 3), which can be destabilized by applied minimal stress to the cell. The study further proposes that the borderline stability of the AAR protein makes it prone to aggregation when expressed in *E. coli* cells for hydrocarbon production and in turn affecting the hydrocarbon production level. Hydrocarbon production levels have been reported to fluctuate with varying soluble levels of AAR^14^.

This highlights the fact that the AAR protein aggregation could be one of the limitations associated with reduced hydrocarbon production, which in the present study could be correlated with the marginal stability of the protein when expressed in *E. coli*. Thus, the outcome of the present study involving the unfolding pathways, determination of thermodynamic stability provides us preliminary information about the stability and mechanistic aspects of the unfolding of a key nodal enzyme involved in biosynthetic hydrocarbon production pathway. Taking into consideration the present studies and further careful investigation in the near future, it would certainly provide much-focused information on improving the stability of the enzyme in question, in turn, to further improve hydrocarbon production.

## Conclusion

The present work investigated the crucial structural aspects of AAR protein associated with its stability and folding, which in-turn reflected on structural integrity of the protein. The protein milieu has been shown to play a significant role in dictating its folding. Apparent standard free energy $\left( {\Delta {\text{G}}_{{{\text{NU}}}}^{{{\text{H}}_{2} {\text{O}}}} } \right)$ elucidated for steady-state unfolding of AAR was indicative of borderline stability of the protein. MD simulation conducted on AAR structure suggested that the ionic solvent KCl mediates structural stabilization and regional alteration towards the binding site of its neighbouring pathway enzyme aldehyde deformylating oxygenase. Based on these evidences, we propose that the marginal stability of AAR are plausible contributing reasons for aggregation propensity and hence low catalytic activity of the enzyme when expressed in *E. coli* for biofuel production.

## Methods

### Expression and purification of AAR

The M15 strain of *E. coli* was transformed with the pQE30AAR plasmid containing codon optimized gene encoding AAR from *Synechococcus elongatus* PCC7942^8^. The transformed *E. coli* cells were grown overnight in LB medium (containing 100 µg/mL Ampicillin) at 37 °C with shaking at 150 rpm to obtain primary culture. The cells were further inoculated from the primary culture into a secondary culture of 1000 mL of LB medium containing ampicillin (100 µg/mL) and cultured at 37 °C with shaking at 150 rpm. Growth of the cells was monitored and the cells were induced with 1.0 mM of IPTG when the OD_600_ reached about 0.8. Post induced cells were cultured for 18 h at 18 °C with shaking at 150 rpm. The cells were subsequently harvested for isolating the expressed AAR protein. A 200 µL of cell culture was aliquoted and centrifuged to collect the cell pellet. The cell pellet was resuspended in SDS-PAGE loading buffer, boiled at 95 °C, and analyzed on 12% SDS-PAGE gel to confirm protein expression.

The harvested cells were further lysed by sonication for the preparation of the soluble AAR. After lysis, the cell lysate was centrifuged at 10,000 rpm for one hour at 4 °C. The supernatant obtained was filtered through 0.45 µm filter (Millipore) before applying to the chromatography column in FPLC unit (BioRad). AAR was purified by a 2-step method which includes metal affinity chromatography using Ni–NTA resin (Qiagen) and Gel Filtration Chromatography (GFC) using HiLoad 16/600 Superdex 200 prep grade column (GE Life Sciences). Fractions containing AAR were identified by SDS-PAGE and were pooled. Protein was stored in 50 mM Tris, 100 mM NaCl, and 1 mM DTT at pH7.4. The protein concentration was determined using an extinction coefficient of 41,940 M^−1^ cm^−1^ at 280 nm.

### Activity assay of AAR

In vitro enzyme assay was carried out to determine the activity of the purified AAR. Assay was performed in 50 mM Tris buffer containing 250 mM KCl, 2 mM MgCl_2_, 10 mM DTT, 1 mg/mL BSA, 1 mM NADPH, 400 µM Palmitoyl CoA, and 5 µM AAR at pH 8.0. The reaction mix was incubated at 37 °C for different time intervals of 0 to 6 h, and an equal volume of ethyl acetate was used for the termination of the reaction. The collected sample was then vortexed at high speed for 10 min and centrifuged. The aqueous phase was analyzed by GC–MS/MS.

### Dynamic light scattering of AAR

A different set of GFC-eluted fractions of AAR were pooled and concentrated to achieve 1 mg/mL concentration of each pooled fraction. Samples were then centrifuged at 10,000 rpm for 30 min at 4 °C. Light scattering measurements were taken using Zetasizer Nano-ZS (Malvern Instruments, Malvern, UK) at a light scattering angle of 90° using 632 nm laser. All studies were conducted at room temperature. AAR-size distributions were obtained using the Malvern Zetasizer software (version 7.12).

### Mass spectrometry-based characterization of AAR

LC–MS analysis of AAR protein was performed using Orbitrap Fusion Lumos Tribrid Mass Spectrometer (Thermo Fischer Scientific, Singapore). Ultra-High Pressure Liquid Chromatography (UHPLC) C8 column (Eclipse plus C8, 2.1 × 150 mm, 5 um, P/N 959701-906, USA) coupled to ESI-Mass Spectrometer was used for the intact mass determination of AAR protein. 500 ng of AAR (prepared in 10 mM ammonium acetate, 0.1% formic acid) was injected into the UHPLC-C8 column. Running conditions were as follows: oven temperature 40 °C, eluent flow rate 0.3 ml/min, overall run time 10 min. Protein is eluted using an increasing gradient of acetonitrile, 0.1% formic acid, and is subjected to ESI–MS. The full ESI–MS (150 to 2000 m/z, 3500 V, Sheath gas 42 arb, Aux gas flow 10 arb, Sweep gas flow 1 arb, S-lens RF 40, Capillary temp 340 °C and Aux gas heater temp 360 °C) scan was obtained and was deconvoluted using Thermo Scientific Biopharma Finder software with the following set parameters: m/z range: 1000–3000, output mass range: 10,000–160,000, mass tolerance: 0.04 Da, Target mass: 80,000 Da, Charge state range: 10–100, choice of peak model: Intact Protein. De-lipidation of AAR was carried out using Shimadzu MAYI-ODS column (5 mm L × 2.0 mm I.D., 50 μm). AAR protein (1 μg) was injected into the ODS column and eluted using 10 mM ammonium acetate buffer within 1.5 min of the elution time monitored by UV-280 nm. The flow rate was kept at 0.6 mL/min and the column temperature was set at 40 °C. The eluted AAR fraction was collected and further injected into the UHPLC C8 column coupled to ESI-mass spectrometer for intact mass determination. MAYI-ODS trapped lipid fraction was collected using gradient method, mobile phase A is 10 mM ammonium formate and mobile phase B, 2-propanol. The %B composition was varied as follows: 5% (0–1 min) → 100% (6–7 min) → 5% (7.01–9 min) at a flow rate of 0.3 ml/min.

### Secondary and tertiary structure determination of AAR

The secondary structure of AAR was determined by recording far-UV CD spectra on JASCO J-815 CD polarimeter at wavelength 200–250 nm in a 1 mm quartz cuvette. The protein concentration was 3.5 μM in buffer containing 50 mM Tris, 100 mM NaCl, 1 mM DTT at pH 7.4. AAR contains six tyrosine and six tryptophan residues, which help in exploiting the intrinsic fluorescence property of the protein to determine the tertiary structure of the protein via Cary Eclipse fluorimeter. The protein was excited at excitation wavelength of 280 nm and 295 nm with slid width of 10 nm and fluorescence emission spectra were recorded from 300 to 400 nm in 1 cm quartz cuvette. The protein concentration used was 3.5 μM in buffer containing 50 mM Tris, 100 mM NaCl, 1 mM DTT at pH 7.4.

### Thermal denaturation and renaturation profile probed by CD

Thermal unfolding studies were carried out for the AAR (3.5 µM) using CD spectroscopy. The proteins were prepared in 50 mM Tris, 100 mM NaCl, 1 mM DTT at pH 7.4. The sample was placed in 1 mm quartz cuvette and was heated from 20 to 90 °C with a heating rate of 1 °C/min controlled by Jasco controllable Peltier PTC-423S/15. For thermal refolding studies, the 90 °C denatured AAR (3.5 µM) solution was gradually cooled to 20 °C with a cooling rate of 1 °C/min. A continuous CD scan at varying wavelength of 210–250 nm was collected for each temperature and the Mean Residual Ellipticity (MRE) values were plotted against the respective temperature. The thermal denaturation profile was analyzed when the obtained set of data was fitted in the two-state model to determine a mid-point of the transition curve using SigmaPlot Version 11.0 software as described in the data analysis section.

### Thermal denaturation and renaturation profile probed by intrinsic fluorescence spectroscopy

For the fluorescence-based thermal denaturation studies, AAR (3.5 µM) in 1 cm quartz cuvette was placed in a thermostatic holder of Cary Eclipse fluorimeter. The holder was maintained at a constant temperature by circulating water from a constant temperature water bath. A step-wise heat denaturation experiment was performed. AAR solution was incubated at different temperatures varying from 20 to 90 °C for 5 min before recording the emission spectra. Each incubated sample was excited at 295 nm and emission spectra were recorded from 300 to 400 nm at all temperatures with slit width of 10 nm. For the step-wise thermal renaturation experiment, AAR solution (3.5 µM) was incubated at 90 °C for 5 min and then gradually cooled. The incubated sample was excited at 295 nm and the intrinsic fluorescence emission spectra were recorded from 300 to 400 nm at different temperatures from 20 to 90 °C.

The relative fluorescence intensity at 340 nm and emission maximal wavelength ƛ_max_ was plotted against the temperature to obtain the thermal denaturation transition curve. The thermal denaturation profile was analyzed when the obtained set of data was fitted in the two-state model to determine a mid-point of the transition curve using SigmaPlot Version 11.0 software as described in the data analysis section.

### Equilibrium unfolding of AAR probed by CD

Denaturant-mediated equilibrium unfolding studies were carried out using urea and GdnHCl as denaturant using CD spectroscopy. Spectra were recorded in the far-UV region (wavelength 200–250 nm) using JASCO J-815 CD polarimeter equipped with JASCO PTC-423S/15 using 1 mm quartz cuvette. The samples were prepared by incubating AAR (3.5 µM) with different concentrations of GdnHCl (0 to 6 M) or urea (0–8 M) for one hour at 25 °C in buffer containing 50 mM Tris, 100 mM NaCl, and 1 mM DTT at pH 7.4. All the experiments were carried out in triplicates. Each spectrum was corrected against its blank. The MRE values were plotted against the denaturant concentrations to obtain an unfolding transition curve. The unfolding transition profile was analyzed when the obtained set of data was fitted in the two/ three-state model to determine a mid-point of the transition curve and steady-state free energy change parameters using SigmaPlot Version 11.0 software as described in the data analysis section.

### Equilibrium unfolding probed by intrinsic fluorescence spectroscopy

Intrinsic fluorescence property was also used to probe the equilibrium unfolding of AAR. The samples were placed in 1 cm quartz cuvette with slit width of 10 nm. The excitation of equilibrated protein samples were done at 295 nm and emission spectra were recorded from 300 to 400 nm. AAR protein (2.0 µM) was incubated with varying denaturant concentrations (0–8 M Urea or 0–6 M GdnHCl) for an hour at 25 °C in buffer containing 50 mM Tris, 100 mM NaCl, and 1 mM DTT at pH 7.4. Blank spectrum at each concentration was taken and subtracted from the respective sample spectrum. All the experiments were carried out in triplicates. The wavelength at which maximum emission was observed (ƛ_max_) and relative fluorescence intensity at 340 nm were plotted against the corresponding denaturant concentration. The relative fluorescence intensity represents fluorescence intensity at 340 nm at a particular concentration minus fluorescence intensity at 340 nm of denaturant max. concentration.

### Data analysis of two-state equilibrium unfolding

The urea-induced equilibrium unfolding data obtained from CD and fluorescence spectroscopy was analyzed by drawing the baseline for the native and unfolded states in the unfolding transition curve which can be further fitted to a two-state model^30^. The proposed equilibrium is1
where K_NU_ is the equilibrium constant for N ⥨ U.

The observed signal of protein from both CD and fluorescence intensity of the protein [S_obs_(c)] at any concentration of denaturant is given by the sum contribution of all the two states as:

2 $${\text{S}}_{{{\text{obs}}}} \left( {\text{c}} \right) = {\text{S}}_{{\text{N}}} {\text{f}}_{{\text{N}}} \left( {\text{c}} \right) + {\text{S}}_{{\text{U}}} {\text{f}}_{{\text{U}}} \left( {\text{c}} \right)$$

f_N_ (c), f_U_ (c) are the fractions of two states at a urea concentration of c and S_N_, and S_U_ are the signal for pure N and U states respectively.

The fractions f_N_, and f_U_ are related to K_NU_ of unfolding transition from N ⥨ U as

3 $${\text{K}}_{{{\text{NU}}}} = {\text{f}}_{{\text{u}}} /{\text{f}}_{{\text{n}}}$$

Hence, are related to corresponding free energy change ΔG_NU_ as follow:

4 $$\Delta {\text{G}}_{{{\text{NU}}}} = - {\text{RT}}\;\ln \;{\text{K}}_{{{\text{NU}}}}$$

where R is gas constant and T is absolute temperature.

It is well-known that the free energy (ΔG_NU_) of unfolded protein at different denaturant concentrations [D] has a linear relation with the denaturant concentration:

5 $$\Delta {\text{G}}_{{{\text{NU}}}}^{{\text{D}}} = \Delta {\text{G}}_{{{\text{NU}}}}^{{{\text{H}}_{2} {\text{O}}}} {-}{\text{m}}\;\left[ {\text{D}} \right]$$

where $\Delta {\text{G}}_{{{\text{NU}}}}^{{{\text{H}}_{2} {\text{O}}}}$ is the free energy of unfolding in absence of denaturant; m is the slope of the transition.

The observed signal of protein by CD and fluorescence intensity is given by

6 $${\text{S}}_{{{\text{obs}}}} \left( {\text{c}} \right) = {\text{S}}_{{\text{N}}} + {\text{S}}_{{\text{U}}} \exp \left[ { - \left( {\Delta {\text{G}}_{{{\text{NU}}}}^{{{\text{H}}_{2} {\text{O}}}} - {\text{m}}_{{{\text{NU}}}} \;{\text{c}}} \right)/{\text{RT}}} \right]/1 + \exp \left[ { - \left( {\Delta {\text{G}}_{{{\text{NU}}}}^{{{\text{H}}_{2} {\text{O}}}} - {\text{m}}_{{{\text{NU}}}} \;{\text{c}}} \right)/{\text{RT}}} \right.$$

In general S_N_ and S_U_ are dependent on the concentration of c, and we assume a linear dependence on the concentration of denaturant (c), as S_N_ = a_1_ + b_1_c, S_U_ = c_1_ + p_1_c; where a_1_, b_1_, c_1_, and p_1_ are constants. a_1_, b_1_, c_1_, p_1_ were calculated from intercept and slope of the baseline of the native and unfolded states. By using all the above equations, the urea-mediated equilibrium unfolding data were analyzed by a method of nonlinear least-squares analysis and thermodynamic parameters of stability were calculated. The data were fitted to this equation using the linear least-square method to obtain the best-fitted values of $\Delta {\text{G}}_{{{\text{NU}}}}^{{{\text{H}}_{2} {\text{O}}}}$ and D_m_. The curve fittings were performed using SigmaPlot Version 11.0 software.

### Data analysis of GdnHCl-mediated equilibrium unfolding

The GdnHCl-induced equilibrium unfolding data obtained from CD and fluorescence spectroscopy was analyzed by drawing the baseline for the native, intermediate, and unfolded states in the unfolding transition curve which can be further fitted to a three-state model^31^. The proposed equilibrium is7
where K_NI_, K_IU_, and K_NU_ are the equilibrium constants for N ⥨ I, I ⥨ U, and N ⥨ U transitions, respectively. The observed signal of protein from both CD and fluorescence intensity of the protein [S_obs_(c)] at any concentration of denaturant is given by the sum contribution of all the three states as:

8 $${\text{S}}_{{{\text{obs}}}} \left( {\text{c}} \right) = {\text{S}}_{{\text{N}}} {\text{f}}_{{\text{N}}} \left( {\text{c}} \right) + {\text{S}}_{{\text{I}}} {\text{f}}_{{\text{I}}} \left( {\text{c}} \right) + {\text{S}}_{{\text{U}}} {\text{f}}_{{\text{U}}} \left( {\text{c}} \right)$$

f_N_ (c), f_I_ (c), f_U_ (c) are the fractions of three states at a GdnHCl concentration of c and S_N_, S_I_, and S_U_ are the signal for pure N, I, and U states respectively. The fractions f_N_, f_I_ and f_U_ are related to K_NI_ and K_NU_ of unfolding transition from N ⥨ I and N ⥨ U respectively, hence are related to corresponding free energy changes ΔG_NI_ and ΔG_NU_, as follows.

9 $$\begin{aligned} {\text{f}}_{{\text{N}}} & = 1/(1 + {\text{K}}_{{{\text{NI}}}} + {\text{K}}_{{{\text{NU}}}} ) = 1/[1 + \exp \, ( - \Delta {\text{G}}_{{{\text{NI}}}} /{\text{RT}}) + \exp ( - \Delta {\text{G}}_{{{\text{NU}}}} /{\text{RT}})] \\ {\text{f}}_{{\text{I}}} & = {\text{K}}_{{{\text{NI}}}} /(1 + {\text{K}}_{{{\text{NI}}}} + {\text{K}}_{{{\text{NU}}}} ) = \exp \, ( - \Delta {\text{G}}_{{{\text{NI}}}} /{\text{RT}})/[1 + \exp ( - \Delta {\text{G}}_{{{\text{NI}}}} /{\text{RT}}) + \exp \, ( - \Delta {\text{G}}_{{{\text{NU}}}} /{\text{RT}})] \\ {\text{f}}_{{\text{U}}} & = {\text{K}}_{{{\text{NU}}}} /(1 + {\text{K}}_{{{\text{NI}}}} + {\text{K}}_{{{\text{NU}}}} ) = \exp \, (\Delta {\text{G}}_{{{\text{NU}}}} /{\text{RT}})/[1 + \exp ( - \Delta {\text{G}}_{{{\text{NI}}}} /{\text{RT}}) + \exp \, ( - \Delta {\text{G}}_{{{\text{NU}}}} /{\text{RT}})] \\ \end{aligned}$$

where R is gas constant and T is the absolute temperature. The free energy changes of unfolding are known to vary linearly with denaturant concentration such that

10 $$\begin{aligned} \Delta {\text{G}}_{{{\text{NI}}}} & = \Delta {\text{G}}_{{{\text{NI}}}}^{{{\text{H}}_{2} {\text{O}}}} - {\text{ m}}_{{{\text{NI}}}} \;{\text{c}} \\ \Delta {\text{G}}_{{{\text{NU}}}} & = \Delta {\text{G}}_{{{\text{NU}}}}^{{{\text{H}}_{2} {\text{O}}}} - {\text{ m}}_{{{\text{NU}}}} \;{\text{c}} \\ \end{aligned}$$

where $\Delta {\text{G}}_{{{\text{NU}}}}^{{{\text{H}}_{2} {\text{O}}}}$ and $\Delta {\text{G}}_{{{\text{NI}}}}^{{{\text{H}}_{2} {\text{O}}}}$ are the ΔG_NU_ and ΔG_NI_ at 0 M GdnHCl respectively and m_NI_ and m_NU_ represent the dependence of respective free energy changes on denaturant concentration (c) and the co-operativity of transition.

So from the Eqs. (8)–(10), S_obs_ (c) is given by:

11 $$\begin{aligned} {\text{Sobs}}\left( {\text{c}} \right) & = {\text{S}}_{{\text{N}}} + {\text{S}}_{{\text{I}}} {\text{exp}}\left[ { - \left( {\Delta {\text{G}}_{{{\text{NI}}}}^{{{\text{H}}_{2} {\text{O}}}} - {\text{m}}_{{{\text{NI}}}} \;{\text{c}}} \right)/{\text{RT}}} \right] + {\text{S}}_{{\text{U}}} {\text{exp}}\left[ { - \left( {\Delta {\text{G}}_{{{\text{NU}}}}^{{{\text{H}}_{2} {\text{O}}}} - {\text{m}}_{{{\text{NU}}}} \;{\text{c}}} \right)/{\text{RT}}} \right]/1 \\ & \quad + \,{\text{exp}}\left[ { - \left( {\Delta {\text{G}}_{{{\text{NI}}}}^{{{\text{H}}_{2} {\text{O}}}} - {\text{m}}_{{{\text{NI}}}} \;{\text{c}}} \right)/{\text{RT}}} \right] + {\text{exp}}\left[ { - \left( {\Delta {\text{G}}_{{{\text{NU}}}}^{{{\text{H}}_{2} {\text{O}}}} - {\text{m}}_{{{\text{NU}}}} \;{\text{c}}} \right)/{\text{RT}}} \right] \\ \end{aligned}$$

In general S_N_, _SI_ and S_U_ are dependent on the concentration of c, and we assume a linear dependence on the concentration of denaturant (c), as S_N_ = a_1_ + b_1_c, S_I_ = c_1_ + p_1_c and S_U_ = e_1_ + g_1_c where a_1_, b_1_, c_1_, p_1_, e_1_ and g_1_ are constants. a_1_, b_1_, c_1_, p_1_, e_1_, g_1_ were calculated from intercept and slope of the baseline of the native, intermediate, and unfolded states. By using all the above equations, the GdnHCl-mediated equilibrium unfolding data were analyzed by a method of nonlinear least-squares analysis and thermodynamic parameters of stability were calculated.

### Size exclusion chromatography of AAR

Size exclusion chromatography of AAR (1.5 mg/mL) in presence or absence of 2.25 M concentration of GdnHCl was carried out using Superdex increase 200 10/300 GL column. Buffer conditions used were 50 mM Tris, 100 mM NaCl, and 1 mM DTT at pH7.4 with or without 2.25 M GdnHCl. 200 μL of sample was injected to the column and absorbance at UV-280 nm was used to monitor the elution profile at 1 mL/min of the flow rate.

### Molecular dynamics (MD) simulations

The crystal structure of AAR was obtained from the PDB entry 6JZU. The obtained structure was prepared before the simulation using UCSF Chimera Version 1.11.2 tool^32^. The molecular dynamics on 298 K and 303 K were performed using AMBER18 software^33,34^ and modelling and data analysis were performed by AmberTools18 suite of programs^33,34^. The parameters and atom-types of hexadecanal ligand were generated through ANTECHAMBER module and charges calculated using AM1-BCC method and the topology and parameter files were constructed using the force field leaprc.ff99SBxildn for the AAR protein. The systems were solvated with water molecules using the 10Ȧ pad of TIP3P water model. Neutralizing counter Na^+^ ions were added first, thereafter based on the required concentrations for the study the Cl^−^ and K^+^ ions were added to obtain respective systems of 0, 125, 250, 500 and 1000 mM of KCl environment. The energy minimization procedure included initial 500 cycles of steep descent (SD) algorithm followed by remaining 19,500 cycles of conjugate gradient algorithm (CG) totaling 20,000 steps. The systems were heated from 0 to 298 K and 303 K over 50 ps with a collision frequency of 2.0 ps^−1^ and weak harmonic restraints of 2 kcal mol^−1^ Å^−2^ on all atoms using Langevin thermostat for temperature regulation. The next step was accompanied by density equilibration (50 ps) and constant pressure equilibration (5 ns). The final production molecular dynamics simulations were performed for 500 ns time interval at 298 K and 303 K temperatures on respective systems of 0, 125, 250, 500 and 1000 mM of KCl and the trajectory was saved at every 2 ps. All molecular simulations were performed using PMEMD module in AMBER18. The time step of 2 fs was used for all MD stages and all atoms involving hydrogen atoms were constrained using the SHAKE algorithm. The CPPTRAJ module^35^ of AmberTools18 was used to perform the RMSD, RMSF and cluster analysis of trajectories. Molecular Mechanics Generalized Born Surface Area (MM-GBSA) based end-point energy calculation was conducted to understand free energy differences between each system. The binding energies of AAR bound with hexadecanal ligand were calculated using 100 snapshots taken from last 200 ns MD trajectory. Cluster representative of the largest cluster was taken from each system at different temperatures from their respective MD trajectories using CPPTRAJ module of AmberTools18 for generating structures for comparison.

## Supplementary Information

Supplementary Information.

## Data Availability

All data generated or analysed during this study are included in this published article and its supplementary information files.

## Abbreviations

AAR: Acyl-acyl carrier protein reductase
ADO: Aldehyde-deformylating oxygenase
ACP: Acyl carrier protein
CD: Circular dichroism
CoA: Coenzyme A
DLS: Dynamic light scattering
GdnHCl: Guanidine hydrochloride
GFC: Gel filtration chromatography
IPTG: Isopropyl β-d-1-thiogalactopyranoside
MS: Mass spectrometry
NADPH: Nicotinamide adenine dinucleotide phosphate
UHPLC: Ultra-high performance liquid chromatography
UV: Ultra violet
