# Supplementary material for: Biophysical and structural studies reveal marginal stability of a crucial hydrocarbon biosynthetic enzyme acyl ACP reductase
Source: Sci Rep. 2021 Jun 8;11:12045. doi: 10.1038/s41598-021-91232-0 (PMC8187606; doi:10.1038/s41598-021-91232-0)
Supplement: Supplementary file 1 — Supplementary Information. [file 41598_2021_91232_MOESM1_ESM.pdf]

Supporting Information

Biophysical and structural studies reveal marginal stability of a crucial hydrocarbon biosynthetic enzyme Acyl ACP Reductase

Ashima Sharma, Tabinda Shakeel, Mayank Gupta, Girish H. Rajacharya, Syed Shams Yazdani*

List of supporting information:

Figure S1. Full images of 12% SDS PAGE gels showing expression and purification of AAR

Figure S2. A. Chromatogram showing AAR elution profile during GFC monitored at 280 nm. B. 12% SDS-PAGE gel showing GFC eluted AAR fractions. C. GFC elution profile of protein standards

Figure S3. DLS of Pooled GFC fractions showing polydisperse state of solution.

Figure S4. LC-MS profile of lipid extract bound to AAR

Figure S5. MD simulation based studies on AAR.

Figure S6: GdnHCl-mediated unfolding of AAR

Figure S7: DLS-based estimation of size distribution by number (%) and volume (%) of different AAR fractions

Figure S8. Urea- mediated equilibrium unfolding of AAR

Figure S9: Characterization of pooled fraction b and c via DLS measurement (A), and individual fraction b and c via CD (B) and intrinsic fluorescence (C) spectroscopy.

Table S1: LC-MS analysis based list of lipid candidates binding to AAR protein.

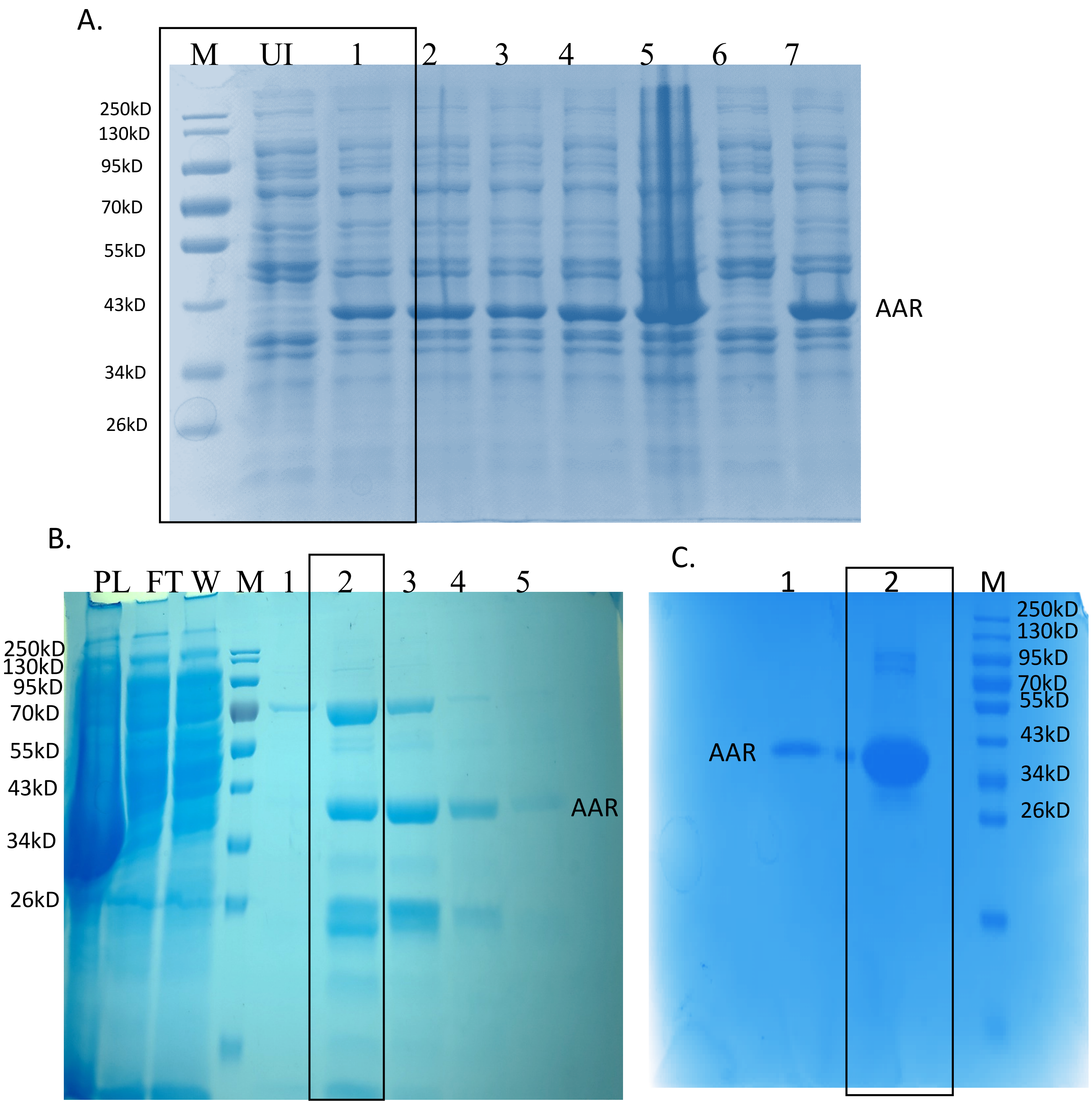

Figure S1. Full images of 12% SDS PAGE gels showing protein expression and purification of AAR. The marked lanes are used in Fig.1 of the main text. (A) Screening of different colonies for AAR expression - gel shows whole cell proteins of uninduced (Lane UI) culture and induced cultures (Lane 1-7). (B) Ni NTA chromatography- Lanes PL, FT, W, and 1-5 represent preload, flow-through, wash, and elute fractions, respectively. (C) Concentrated AAR after GFC purification, Lanes 1-2 represent fractions of finally purified AAR fractions.

A.

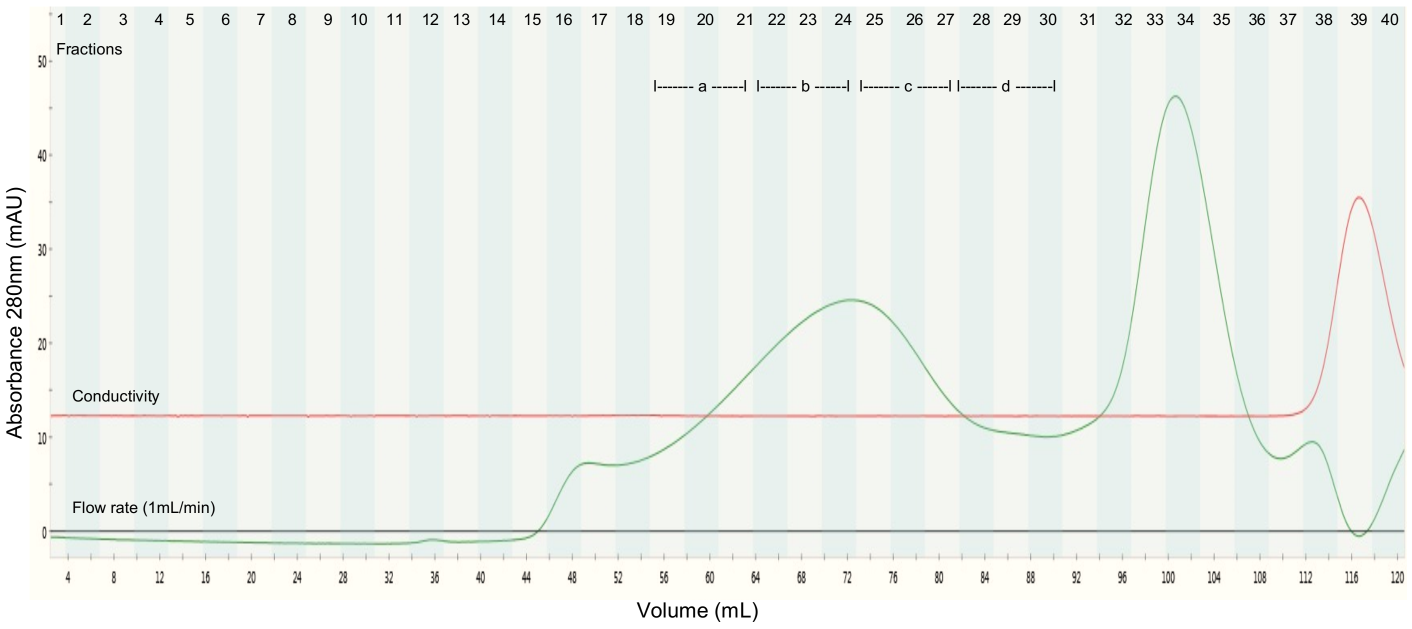

Fractions
1 2 3 4 5 6 7 8 9 10 11 12 13 14 15 16 17 18 19 20 21 22 23 24 25 26 27 28 29 30 31 32 33 34 35 36 37 38 39 40
I------- a ------I I------- b ------I I------- c ------I I------- d -------I
Conductivity
Flow rate (1mL/min)
Absorbance 280nm (mAU)
0 10 20 30 40 50
4 8 12 16 20 24 28 32 36 40 44 48 52 56 60 64 68 72 76 80 84 88 92 96 100 104 108 112 116 120
Volume (mL)

B.

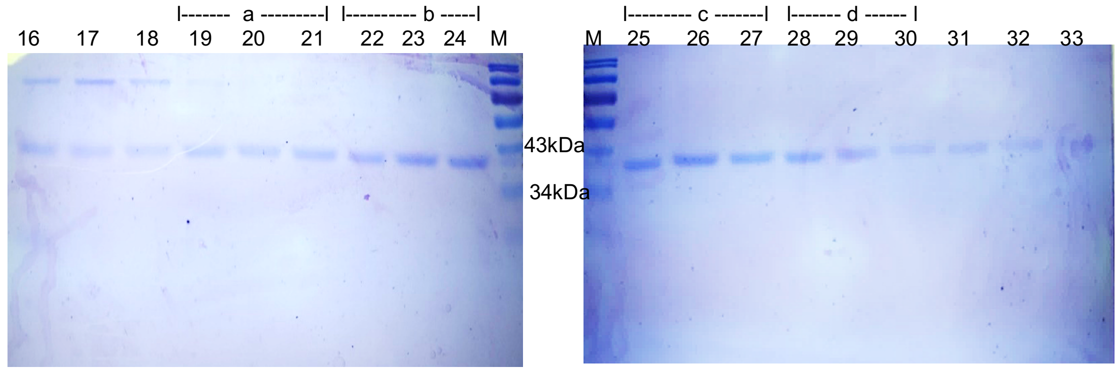

I------- a ---------I I---------- b -----I
16 17 18 19 20 21 22 23 24 M
43kDa
34kDa
I--------- c -------I I------- d ------ I
M 25 26 27 28 29 30 31 32 33

C.

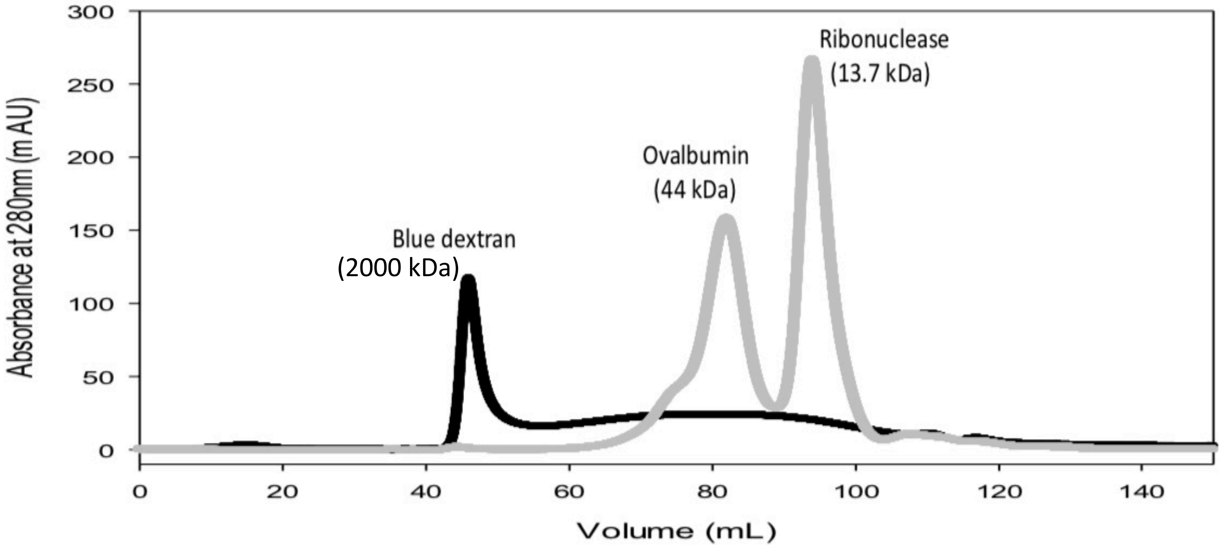

Blue dextran
(2000 kDa)
Ovalbumin
(44 kDa)
Ribonuclease
(13.7 kDa)
Absorbance at 280nm (m AU)
0 50 100 150 200 250 300
0 20 40 60 80 100 120 140
Volume (mL)

Figure S2. A. Chromatogram showing AAR elution profile during GFC monitored at 280 nm. a, b, c, and d represent pooled fraction 19-21, 22-24, 25-27, and 28-30 respectively. B. 12% SDS-PAGE gel showing GFC eluted AAR fractions. Lane 16-33 represents the AAR fractions corresponding to the chromatogram, Lane M, medium molecular weight protein marker. C. GFC elution profile of protein standards. The retention volumes for Blue dextran, Ovalbumin and Ribonuclease are 46.6 ml, 82.5 ml and 94.6 ml, respectively.

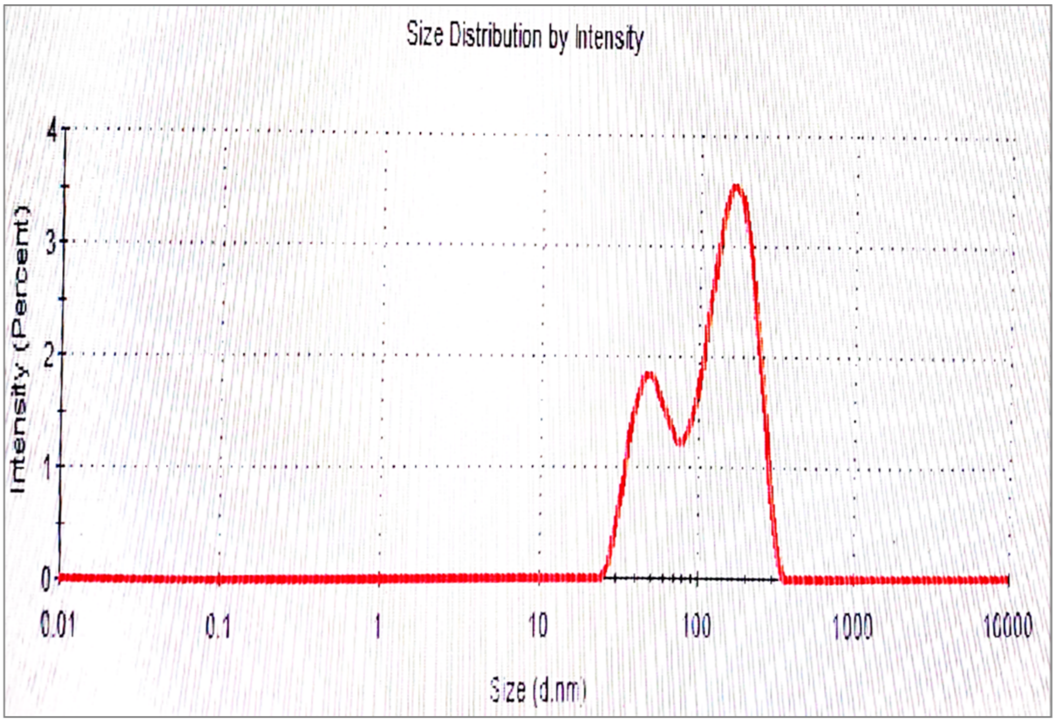

			Size (d.nm):	% Intensity:	St Dev (d.nm):
Z-Average (d.nm):	98.41	Peak 1:	165.1	69.6	53.81
PdI:	0.385	Peak 2:	51.26	30.4	13.33
Intercept:	0.912	Peak 3:	0.000	0.0	0.000

Figure S3. DLS of Pooled GFC fractions showing polydisperse state of solution.

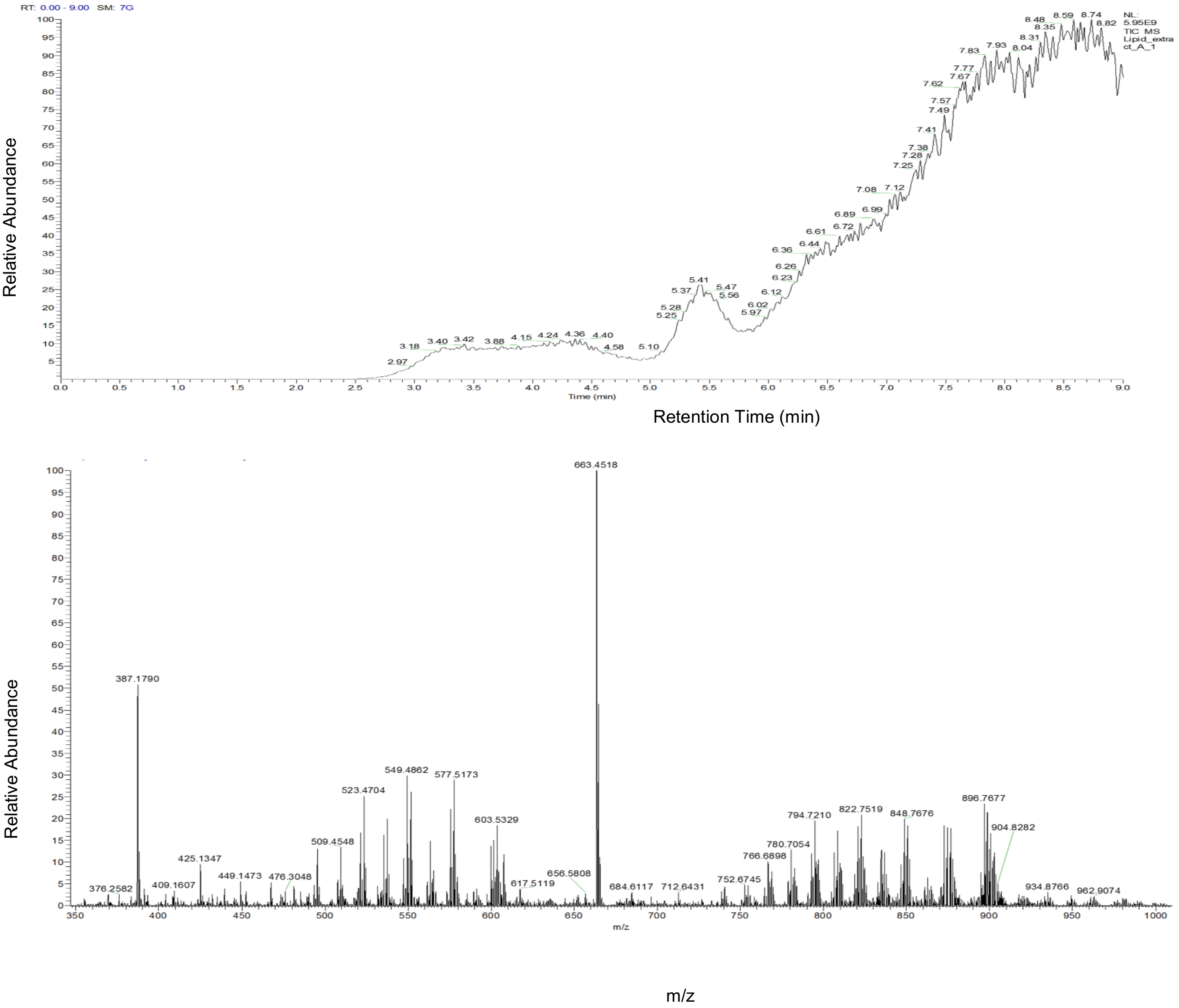

Figure S4: LC-MS profile of lipid extract bound to AAR. A, Fractionation profile of lipid extract. B, Full ESI-MS scan in positive ion mode (300-1000) m/z R_t: 0.2- 8.95 min.

A.

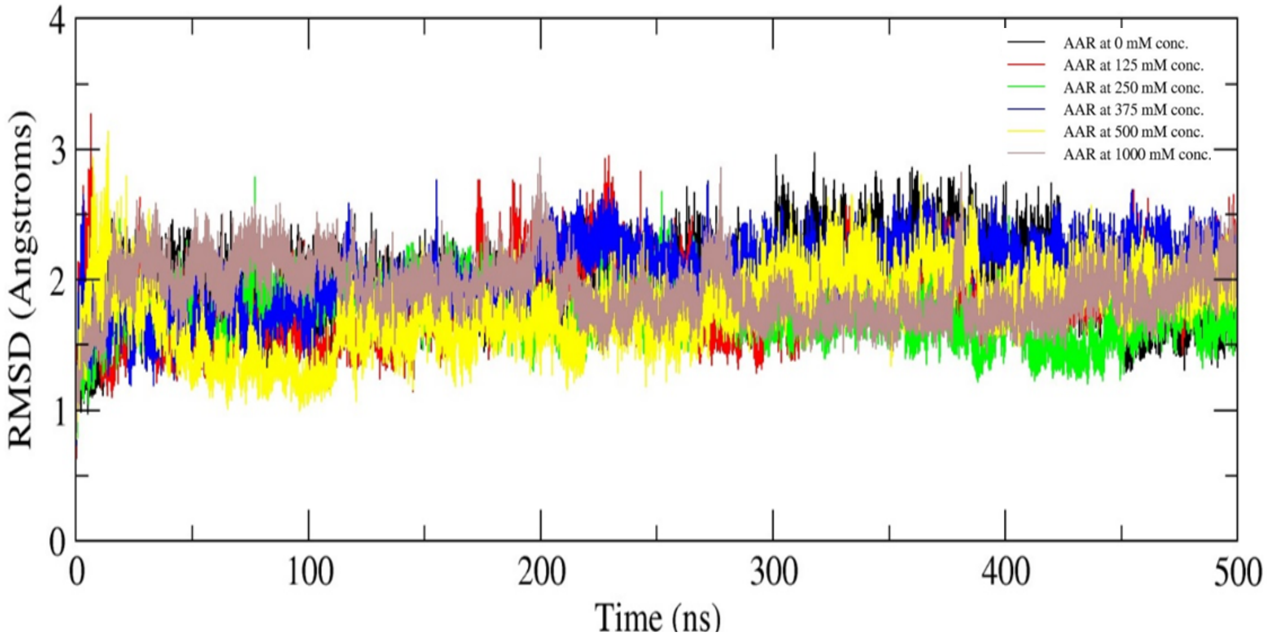

B.

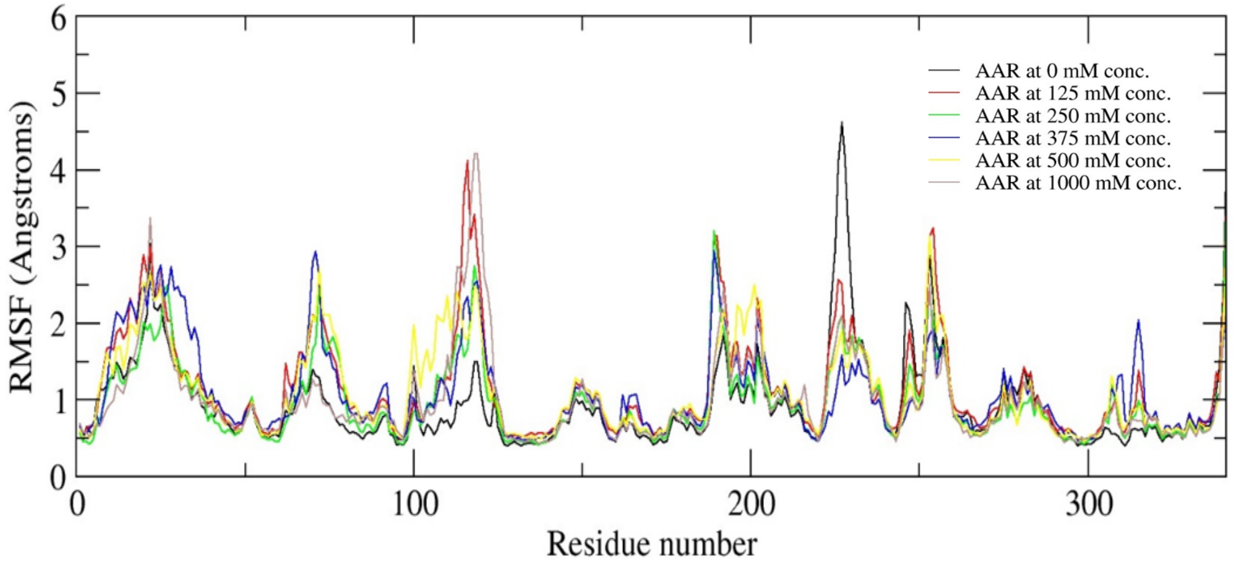

Figure S5. MD simulation-based studies on AAR. A, Average root mean square deviation (RMSD) in Å with reference to the initial structure used in the simulation. **B,** Average root mean square fluctuation (RMSF) in Å for each amino acid of AAR protein.

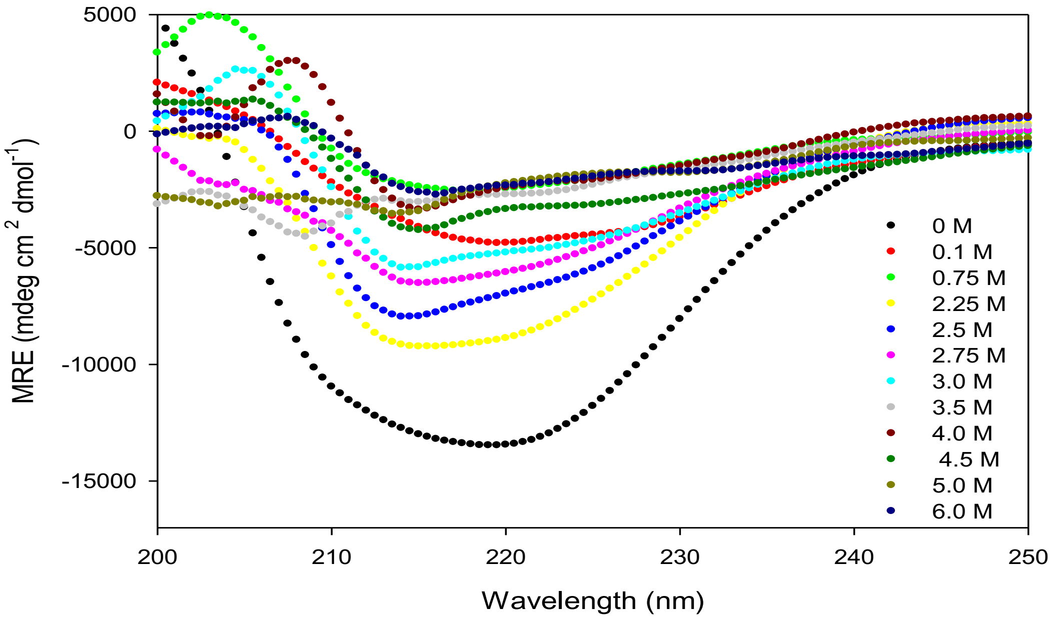

A.

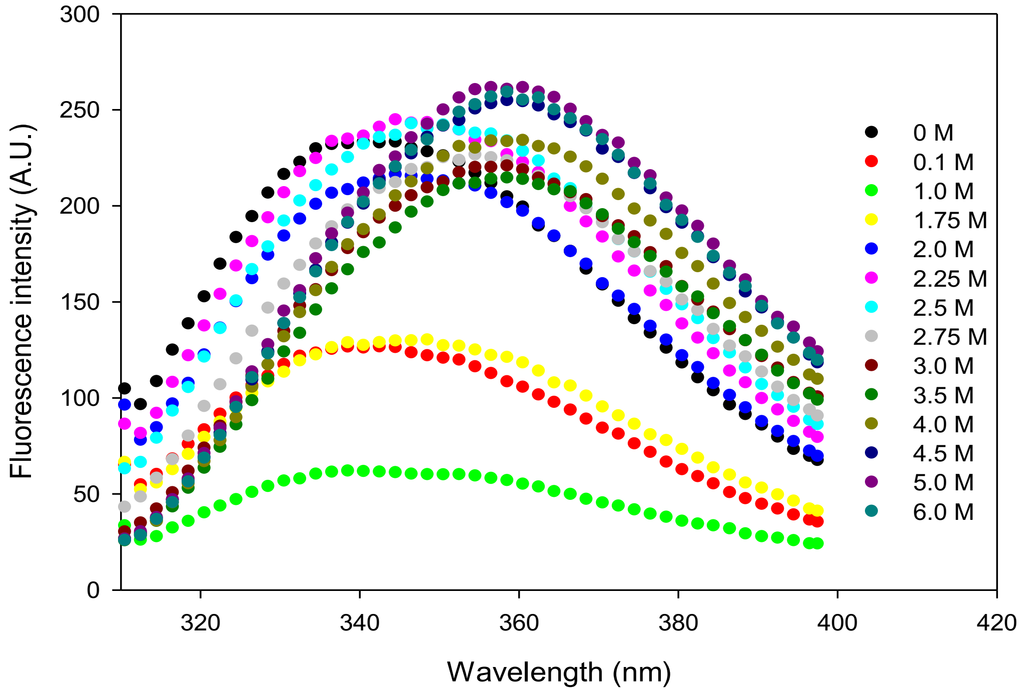

B.

Figure S6. GdnHCl-mediated equilibrium unfolding of AAR probed by CD spectroscopy (A) and intrinsic fluorescence spectroscopy (B).

Z-Average (d.nm): 51.57
PdI: 0.164
Intercept: 0.955
Result quality : Good

	Size (d.nm):	% Volume:	St Dev (d.nm):
Peak 1:	44.93	99.3	8.921
Peak 2:	3707	0.7	1020
Peak 3:	0.000	0.0	0.000

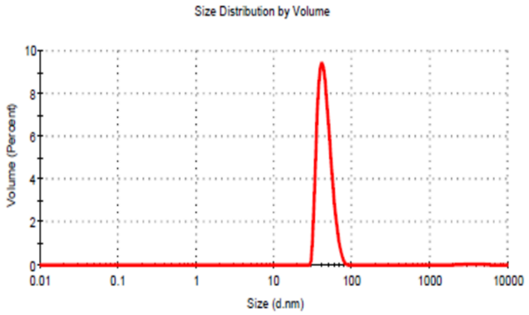

Z-Average (d.nm): 51.57
PdI: 0.164
Intercept: 0.955
Result quality : Good

	Size (d.nm):	% Number:	St Dev (d.n..
Peak 1:	40.90	100.0	7.025
Peak 2:	0.000	0.0	0.000
Peak 3:	0.000	0.0	0.000

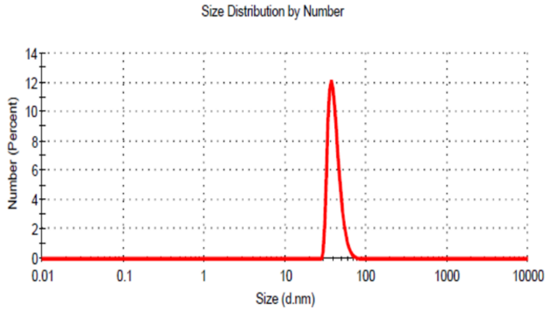

Fraction a

Z-Average (d.nm): 45.33
PdI: 0.146
Intercept: 0.928
Result quality : Good

	Size (d.nm):	% Volume:	St Dev (d.nm):
Peak 1:	36.01	100.0	12.23
Peak 2:	0.000	0.0	0.000
Peak 3:	0.000	0.0	0.000

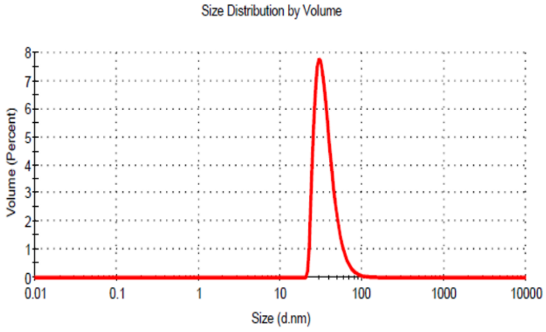

Z-Average (d.nm): 45.33
PdI: 0.146
Intercept: 0.928
Result quality : Good

	Size (d.nm):	% Number:	St Dev (d.n..
Peak 1:	30.36	100.0	6.457
Peak 2:	0.000	0.0	0.000
Peak 3:	0.000	0.0	0.000

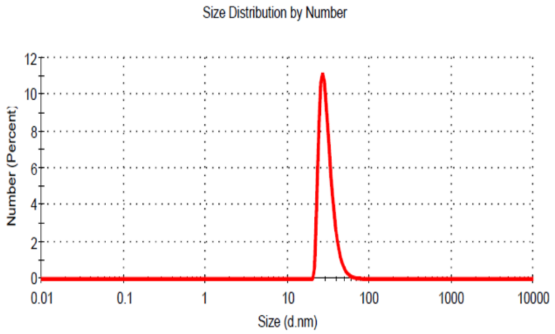

Fraction b

Z-Average (d.nm): 36.38
PdI: 0.192
Intercept: 0.953
Result quality : Good

	Size (d.nm):	% Volume:	St Dev (d.nm):
Peak 1:	30.35	99.9	7.084
Peak 2:	507.0	0.1	172.1
Peak 3:	0.000	0.0	0.000

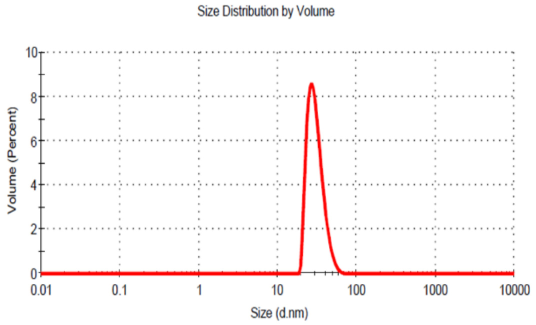

Z-Average (d.nm): 36.38
PdI: 0.192
Intercept: 0.953
Result quality : Good

	Size (d.nm):	% Number:	St Dev (d.n...
Peak 1:	26.91	100.0	5.111
Peak 2:	0.000	0.0	0.000
Peak 3:	0.000	0.0	0.000

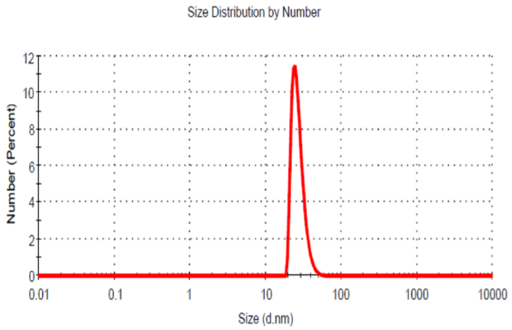

Fraction c

Figure S7: DLS-based estimation of size distribution by number (%) and volume (%) of different AAR fractions

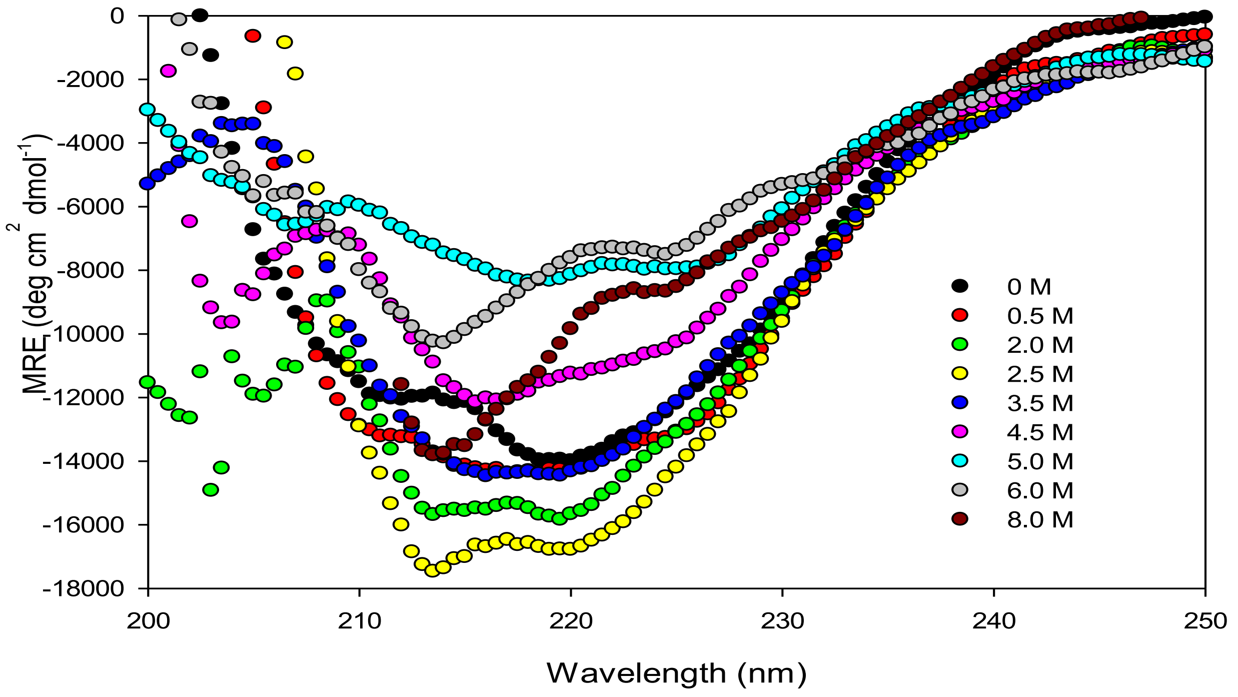

0
-2000
-4000
-6000
-8000
-10000
-12000
-14000
-16000
-18000
MRE (deg cm^2 dmol^{-1})
200
210
220
230
240
250
Wavelength (nm)
0 M
0.5 M
2.0 M
2.5 M
3.5 M
4.5 M
5.0 M
6.0 M
8.0 M

A.

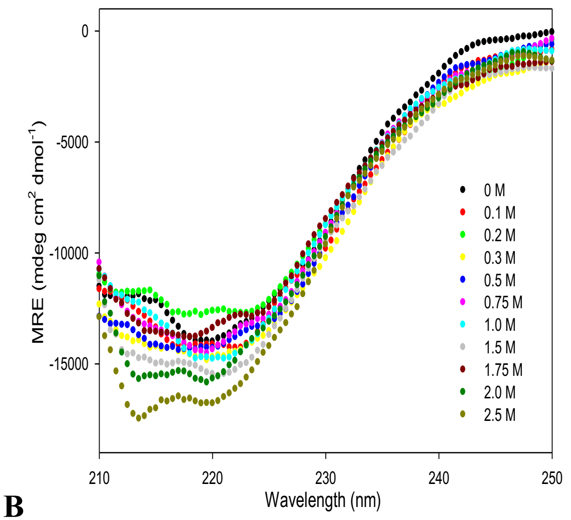

0
-5000
-10000
-15000
MRE (mdeg cm^2 dmol^{-1})
210
220
230
240
250
Wavelength (nm)
0 M
0.1 M
0.2 M
0.3 M
0.5 M
0.75 M
1.0 M
1.5 M
1.75 M
2.0 M
2.5 M

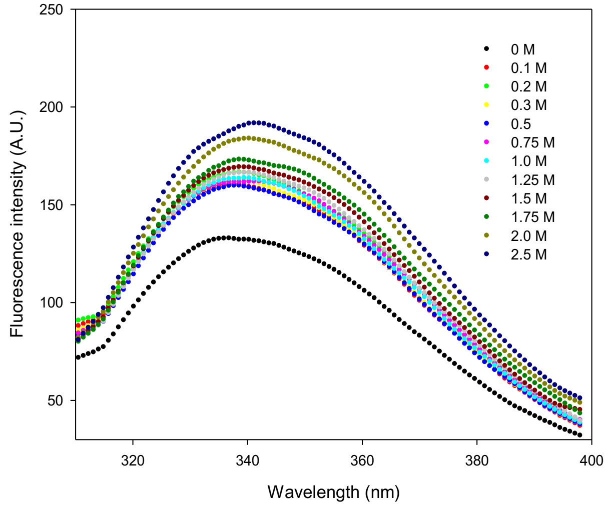

250
200
150
100
50
Fluorescence intensity (A.U.)
320
340
360
380
400
Wavelength (nm)
0 M
0.1 M
0.2 M
0.3 M
0.5
0.75 M
1.0 M
1.25 M
1.5 M
1.75 M
2.0 M
2.5 M

B

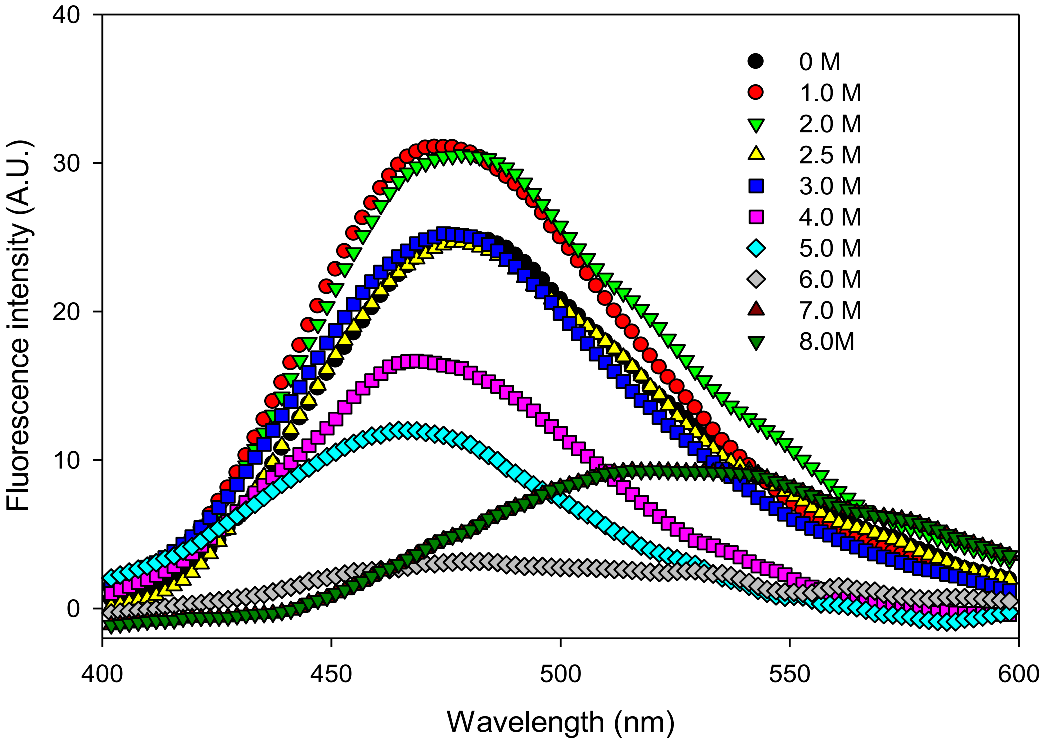

C.

Figure S8. Urea- mediated equilibrium unfolding of AAR. A, Graph showing CD spectra (200-250 nm) of AAR when subjected to different urea concentration 0-8 M. **B**, Graphs showing spectra (210-250 nm) of AAR when subjected to different urea concentration 0- 2.5 M probed by CD spectroscopy and intrinsic fluorescence spectroscopy, respectively. **C**, ANS fluorescence emission spectra in the presence of AAR at various urea (0-8 M) concentrations.

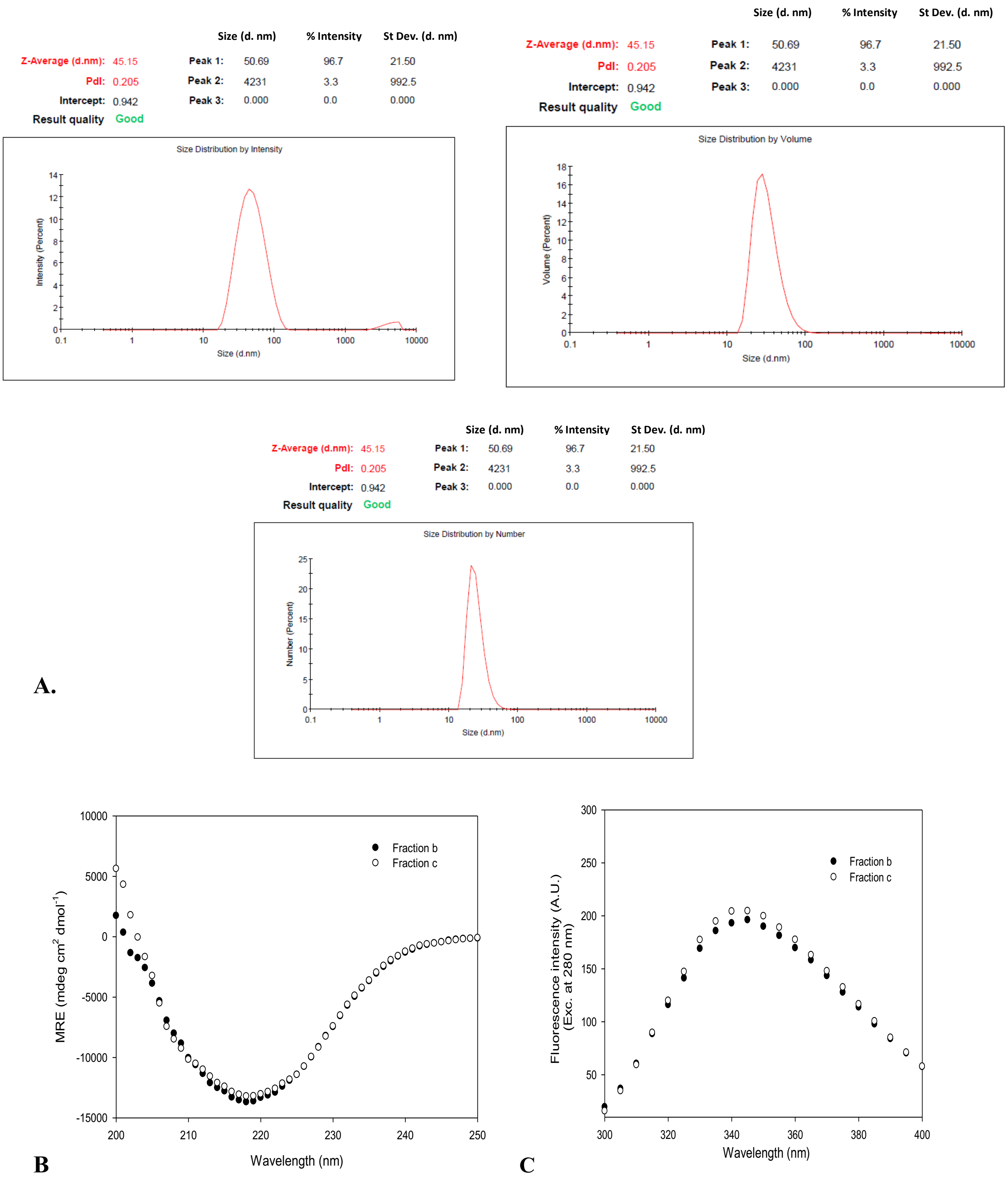

Figure S9: Characterization of pooled fraction b and c via DLS measurement (A), and individual fraction b and c via CD (B) and intrinsic fluorescence (C) spectroscopy.

Table S1: LC-MS analysis based list of lipid candidates binding to AAR protein. The table outlines lipid compound ID, the retention time (RT), m/z value, mass error given in parts per million [ppm]).

Compound ID	Formula	Observed m/z	Calculated m/z	R_t	Error (ppm)
**Fatty acids**					
FA(30:5)+H	C30 H51 O2	443.3875	443.3884	7.18	-2.02
FA(37:6)+H	C37 H63 O2	539.4847	539.4823	7.42	4.6
FA(31:6)+H	C 31 H51 O2	455.3876	455.3884	7.72	-1.62
FA(26:5)+H	C26 H43 O2	387.3239	387.3258	8.59	-4.68
**Hydroxy fatty acids**					
OAHFA(26:0)+H	C26 H51 O4	427.3768	427.3782	1.24	-3.28
OAHFA(55:5)+H	C55 H99 O4	823.7549	823.7538	5.69	1.31
OAHFA(37:1)+H	C37 H71 O4	579.5342	579.5347	5.78	-0.82
OAHFA(54:5)+H	C54 H97 O4	809.7402	809.7381	5.83	2.59
OAHFA(59:8)+H	C59 H101 O4	873.771	873.7694	5.83	1.76
OAHFA(55:6)+H	C55 H97 O4	821.738	821.7381	5.84	-0.11
OAHFA(57:7)+H	C57 H99 O4	847.7555	847.7538	6.07	2.01
OAHFA(39:2)+H	C39 H73 O4	605.5482	605.5503	6.1	-3.51
OAHFA(56:7)+H	C56 H97 O4	833.7402	833.7381	6.23	2.48
OAHFA(56:5)+H	C56 H101 O4	837.771	837.7694	6.23	1.81
OAHFA(51:5)+H	C51 H91 O4	767.693	767.6912	6.33	2.41
OAHFA(36:1)+H	C36 H69 O4	565.5172	565.519	6.44	-3.22
OAHFA(53:6)+H	C53 H93 O4	793.7084	793.7068	6.46	1.99
OAHFA(53:4)+H	C53 H97 O4	797.7386	797.7381	6.46	0.63
OAHFA(54:7)+H	C54 H93 O4	805.7078	805.7068	6.46	1.23
OAHFA(61:4)+H	C61 H113 O4	909.8673	909.8633	6.64	4.3
OAHFA(53:7)+H	C53 H91 O4	791.6933	791.6912	6.75	2.71
OAHFA(50:5)+H	C50 H89 O4	753.6772	753.6755	6.81	2.26
OAHFA(35:1)+H	C35 H67 O4	551.5018	551.5034	6.86	-2.92
OAHFA(44:1)+H	C44 H85 O4	677.6421	677.6442	6.9	-3.19
OAHFA(47:4)+H	C47 H85 O4	713.6457	713.6442	6.93	1.99
OAHFA(50:12)+H	C50 H75 O4	739.5638	739.566	7	-2.99
OAHFA(51:4)+H	C51 H93 O4	769.7083	769.7068	7.04	1.95
OAHFA(60:4)+H	C60 H111 O4	895.8494	895.8477	7.47	1.95
OAHFA(56:6)+H	C56 H99 O4	835.7513	835.7538	7.56	-2.97
OAHFA(61:7)+H	C61 H107 O4	903.8175	903.8164	7.6	1.29
OAHFA(52:4)+H	C52 H95 O4	783.7242	783.7225	7.64	2.16
OAHFA(59:7)+H	C59 H103 O4	875.7866	875.7851	7.65	1.78
OAHFA(60:10)+H	C60 H99 O4	883.7512	883.7538	7.65	-2.91

OAHFA(31:2)+H	C31 H57 O4	493.423	493.4251	7.68	-4.27
OAHFA(56:10)+H	C56 H91 O4	827.6941	827.6912	7.68	3.54
OAHFA(51:2)+H	C51 H97 O4	773.7419	773.7381	7.74	4.92
OAHFA(38:2)+H	C38 H71 O4	591.533	591.5347	7.83	-2.92
OAHFA(35:4)+H	C35 H61 O4	545.4548	545.4564	7.88	-2.96
OAHFA(34:4)+H	C34 H59 O4	531.4392	531.4408	7.89	-3.03
OAHFA(28:1)+H	C28 H53 O4	453.3946	453.3938	7.96	1.68
OAHFA(60:8)+H	C60 H103 O4	887.7811	887.7851	8.02	-4.49
OAHFA(29:1)+H	C29 H55 O4	467.408	467.4095	8.04	-3.27
OAHFA(57:8)+H	C57 H97 O4	845.742	845.7381	8.14	4.57
OAHFA(37:2)+H	C37 H69 O4	577.5172	577.519	8.18	-3.17
OAHFA(50:4)+H	C50 H91 O4	755.6936	755.6912	8.24	3.17
OAHFA(30:2)+H	C30 H55 O4	479.4074	479.4095	8.35	-4.43
OAHFA(30:1)+H	C30 H57 O4	481.4236	481.4251	8.35	-3.20
OAHFA(39:4)+H	C39 H69 O4	601.5174	601.519	8.35	-2.79
OAHFA(39:7)+H	C39 H63 O4	595.4713	595.4721	8.4	-1.34
OAHFA(55:8)+H	C55 H93 O4	817.7078	817.7068	8.44	1.16
OAHFA(32:2)+H	C32 H59 O4	507.4392	507.4408	8.47	-3.20
OAHFA(34:6)+H	C34 H55 O4	527.4107	527.4095	8.48	2.38
OAHFA(49:4)+H	C49 H89 O4	741.6779	741.6755	8.48	3.19
OAHFA(47:6)+H	C47 H81 O4	709.6146	709.6129	8.52	2.36
OAHFA(49:11)+H	C49 H75 O4	727.5656	727.566	8.52	-0.49
OAHFA(41:5)+H	C41 H71 O4	627.534	627.5347	8.55	-1.09
OAHFA(60:6)+H	C60 H107 O4	891.8179	891.8164	8.55	1.73
OAHFA(60:7)+H	C60 H105 O4	889.8024	889.8007	8.61	1.92
OAHFA(32:1)+H	C32 H61 O4	509.4548	509.4564	8.64	-3.23
OAHFA(33:3)+H	C33 H59 O4	519.4392	519.4408	8.64	-3.05
OAHFA(33:2)+H	C33 H61 O4	521.4548	521.4564	8.64	-3.05
OAHFA(33:1)+H	C33 H63 O4	523.4704	523.4721	8.64	-3.22
OAHFA(34:2)+H	C34 H63 O4	535.4704	535.4721	8.64	-3.08
OAHFA(34:1)+H	C34 H65 O4	537.486	537.4877	8.64	-3.14
OAHFA(35:3)+H	C35 H63 O4	547.4705	547.4721	8.64	-2.91
OAHFA(35:2)+H	C35 H65 O4	549.4861	549.4877	8.64	-3.05
OAHFA(36:2)+H	C36 H67 O4	563.5017	563.5034	8.64	-3.08
OAHFA(37:4)+H	C37 H65 O4	573.4861	573.4877	8.64	-2.80
OAHFA(38:4)+H	C38 H67 O4	587.5025	587.5034	8.64	-1.45
OAHFA(39:6)+H	C39 H65 O4	597.4861	597.4877	8.64	-2.69
OAHFA(45:5)+H	C45 H79 O4	683.5997	683.5973	8.64	3.48
OAHFA(31:1)+H	C31 H59 O4	495.4392	495.4408	8.67	-3.13
OAHFA(36:3)+H	C36 H65 O4	561.486	561.4877	8.67	-3.01
OAHFA(38:3)+H	C38 H69 O4	589.5173	589.519	8.67	-2.94
OAHFA(39:5)+H	C39 H67 O4	599.5018	599.5034	8.67	-2.66

OAHFA(42:2)+H	C42 H79 O4	647.5956	647.5973	8.67	-2.59
OAHFA(58:10)+H	C58 H95 O4	855.7251	855.7225	8.71	3.04
OAHFA(39:3)+H	C39 H71 O4	603.5329	603.5347	8.72	-3.02
OAHFA(41:3)+H	C41 H75 O4	631.5643	631.566	8.72	-2.59
OAHFA(34:3)+H	C34 H61 O4	533.4548	533.4564	8.73	-3.03
OAHFA(48:2)+H	C48 H91 O4	731.6886	731.6912	8.73	-3.52
OAHFA(51:7)+H	C51 H87 O4	763.662	763.6599	8.74	2.78
OAHFA(46:2)+H	C46 H87 O4	703.6578	703.6599	8.76	-2.97
OAHFA(43:2)+H	C43 H81 O4	661.6111	661.6129	8.8	-2.76
OAHFA(47:3)+H	C47 H87 O4	715.6576	715.6599	8.8	-3.18
OAHFA(61:5)+H	C61 H111 O4	907.8455	907.8477	8.81	-2.36
OAHFA(42:3)+H	C42 H77 O4	645.581	645.5816	8.82	-0.94
OAHFA(43:1)+H	C43 H83 O4	663.6266	663.6286	8.82	-3
OAHFA(44:2)+H	C44 H83 O4	675.6265	675.6286	8.82	-3.05
OAHFA(45:2)+H	C45 H85 O4	689.6423	689.6442	8.82	-2.77
OAHFA(58:4)+H	C58 H107 O4	867.8174	867.8164	8.83	1.16
OAHFA(52:1)+H	C52 H101 O4	789.7683	789.7694	8.85	-1.39
OAHFA(60:5)+H	C60 H109 O4	893.8336	893.832	8.85	1.80
OAHFA(56:4)+H	C56 H103 O4	839.786	839.7851	8.86	1.05
OAHFA(58:8)+H	C58 H99 O4	859.7561	859.7538	8.86	2.68
OAHFA(59:6)+H	C59 H105 O4	877.8017	877.8007	8.86	1.11
OAHFA(61:8)+H	C61 H105 O4	901.8015	901.8007	8.86	0.86
OAHFA(45:4)+H	C45 H81 O4	685.6149	685.6129	8.88	2.8
OAHFA(43:4)+H	C43 H77 O4	657.5811	657.5816	8.89	-0.82
OAHFA(58:5)+H	C58 H105 O4	865.802	865.8007	8.89	1.41
OAHFA(42:1)+H	C42 H81 O4	649.611	649.6129	8.91	-2.92
OAHFA(47:5)+H	C47 H83 O4	711.6308	711.6286	8.91	3.15
OAHFA(57:5)+H	C57 H103 O4	851.7863	851.7851	8.91	1.44
OAHFA(41:4)+H	C41 H73 O4	629.5483	629.5503	8.94	-3.31
OAHFA(49:6)+H	C49 H85 O4	737.6462	737.6442	8.98	2.71
OAHFA(41:1)+H	C41 H79 O4	635.5953	635.5973	8.99	-3.06
OAHFA(37:3)+H	C37 H67 O4	575.5018	575.5034	9	-2.82
OAHFA(40:1)+H	C40 H77 O4	621.5797	621.5816	9	-3.06
**Prenol lipid**					
Co(Q8)+H	C49 H75 O4	727.5656	727.566	8.52	-0.49
